# Tracing glazed ware production in Islamic Sicily: macroscopic, petrographic, and geochemical evidence

**DOI:** 10.1007/s12520-026-02550-1

**Published:** 2026-09-02

**Authors:** V. Sacco, P. S. Quinn, C. Fenwick

**Affiliations:** Institute of Archaeology, UCL, 31-34 Gordon Square, London, WC1H0PY UK

**Keywords:** Glazed pottery, Ceramic petrography, Geochemistry, Islamic pottery, Islamic Sicily, Medieval Tunisia

## Abstract

This study presents the first large-scale integrated archaeometric investigation of the fabrics of Islamic-period glazed wares produced and consumed in Sicily between the late 9th and 12th centuries. Through the integration of macroscopic analysis, thin-section petrography, and geochemistry, this study refines the characterisation of Sicilian glazed ware fabrics and strengthens their distinction from ceramics produced in Ifriqiya. A total of 188 samples have been analysed from five Sicilian sites: Palermo, Castello della Pietra, Piazza Armerina, Agrigento and Syracuse. The study compares these materials with previously established petrographic fabrics, with the main aim of testing and refining those attributed to specific production centres, and further characterising their compositional variability. Palermo is the most clearly defined centre of production, characterised by the consistent use of the well-known ‘Ficarazzi’ clay source and can be distinguished from other Sicilian manufacturing centres. Workshops at Agrigento, Syracuse, Piazza Armerina, and Mazara del Vallo, where production of glazed ware commenced from the late 10th century, are characterised and interpreted in terms of their likely clay sources. This study also demonstrates that Sicilian products can generally be successfully distinguished from contemporaneous ceramics likely originating from North Africa although some samples attributed to Mazara and Piazza Armerina show compositional affinities that complicate their distinction. While each Sicilian production centre utilised specific local raw materials and their own recipes, certain technological features, including high-temperature firing and salt-white surface treatments on iron-rich fabrics, indicate shared technical knowledge in late 9th – 11th centuries glaze ware production between Sicily and Ifriqiya.

**Supplementary Information:**

The online version contains supplementary material available at https://doi.org/10.1007/s12520-026-02550-1.

## Introduction

In 827 AD, the Aghlabids of Ifriqiya (modern Tunisia and eastern Algeria) launched their conquest of Byzantine Sicily and over the next century and a half the island was gradually integrated into the broader Islamic world. Conquest was followed by significant political, administrative, and economic transformations in the western part of the island, particularly in the new capital, Palermo (Nef 2013; Molinari 2019; 2020). The eastern part remained under Byzantine rule until the later 10th century, when it was conquered by the Fatimid Caliphate, which retained control until the Norman conquest in the second half of the 11th century.

The integration of Sicily into the Islamic world reconfigured ceramic production and consumption, through the introduction of new raw materials, manufacturing techniques, and specialized craftsmanship. As in other domains, the pace and extent of change was uneven: Palermo experienced the earliest and most significant transformations in administrative and economic systems, as well as in urban development and material culture (Nef 2013), reflecting its role as the Aghlabid capital (Sacco 2024; Sacco–Wickham in press; Wickham 2023), while transformations occurred more gradually elsewhere on the island. The introduction of glaze technology was particularly transformative. Glaze was introduced in the late 9th century in Palermo, likely through the mobility of artisans and/or technology transfer from nearby Ifriqiya. The production of glazed ceramics in the two regions shares notable similarities in styles and consumption patterns (Gragueb Chatti et al. 2019; Sacco et al. in press). New decorative styles and vessel forms appeared, which were clearly inspired by broader Islamic models but at the same time reinterpreted locally to suit regional preferences and available raw materials, reflecting shifting consumer demand and changing everyday practices (Sacco 2024).

For nearly a century, Palermo remained the island’s sole centre of glazed ware production, supplying a relatively modest local market that only began to expand in the mid-10th century (Sacco 2024: 251, Sacco–Wickham in press). By the late 10th century, other new workshops had emerged across Sicily, challenging Palermo’s dominance in glazed ceramic production. These developments transformed both production and patterns of tableware consumption, shifting from a single centre to a polycentric model while gradually promoting greater standardisation in forms and decoration. This process eventually influenced practices across the Italian peninsula, as the technology spread from the late 11th to early 12th century and continued through the *Regnum Siciliae* during the 12th century (Sacco–Wickham in press). The Sicilian trajectory differs markedly from that of Ifriqiya, where multiple centres of production operated from an early stage and glazed tableware was already widely distributed by the late ninth century, with Kairouan as the principal production centre (Gragueb Chatti et al. 2019; Occari et al. 2025; Sacco et al. in press; Salinas et al. 2022).

Glazed wares have dominated scholarship on medieval ceramics in Sicily; however, many unanswered questions persist. Due to their similarities in forms and decoration, Sicilian products have often been confused with those from Ifriqiya and broadly labelled as *Siculo-Maghrebin*. Furthermore, the reliance on vague typological labels such as ‘Raqqada type’, ‘pavoncella type’ or dynastic classifications, has obscured internal developments and regional specificities, creating the misleading impression that ceramic traditions remained unchanged for long period of time. While scholars working on ceramics from Ifriqiya continue to face challenges in establishing reliable chronological indicators, the situation in Sicily is gradually improving, thanks to a growing number of specialized studies and increasingly refined chrono-typologies (e.g., Alfano 2019; Arcif–Bagnera 2014, 2018; Ardizzone et al. 2014; Cacciaguerra–Sacco 2022; Meo 2021, 2023; Messina et al. 2018; Sacco 2024).

This paper examines glazed wares from five of the six known production centres in Sicily (Palermo, Mazara, Piazza Armerina, Syracuse and Agrigento) as well as contemporaneous imported glazed wares from Ifriqiya, aiming to characterise the products of the Sicilian workshops and distinguish them from Ifriqiya imports, whose stylistic similarities have long caused confusion. It employs a combination of macroscopic classification, thin-section petrography, and instrumental geochemistry to test and refine traditional visual classifications and previously defined paste groups. By refining existing data and identifying the petrographic and geochemical signatures of the different centres of production, the study lays the groundwork for future research on the provenance, technological choice and selection of raw materials for glazed ware fabrics.

### Locating production centres: evidence and methods for glazed wares in Sicily and Ifriqiya

**Fig. 1:**
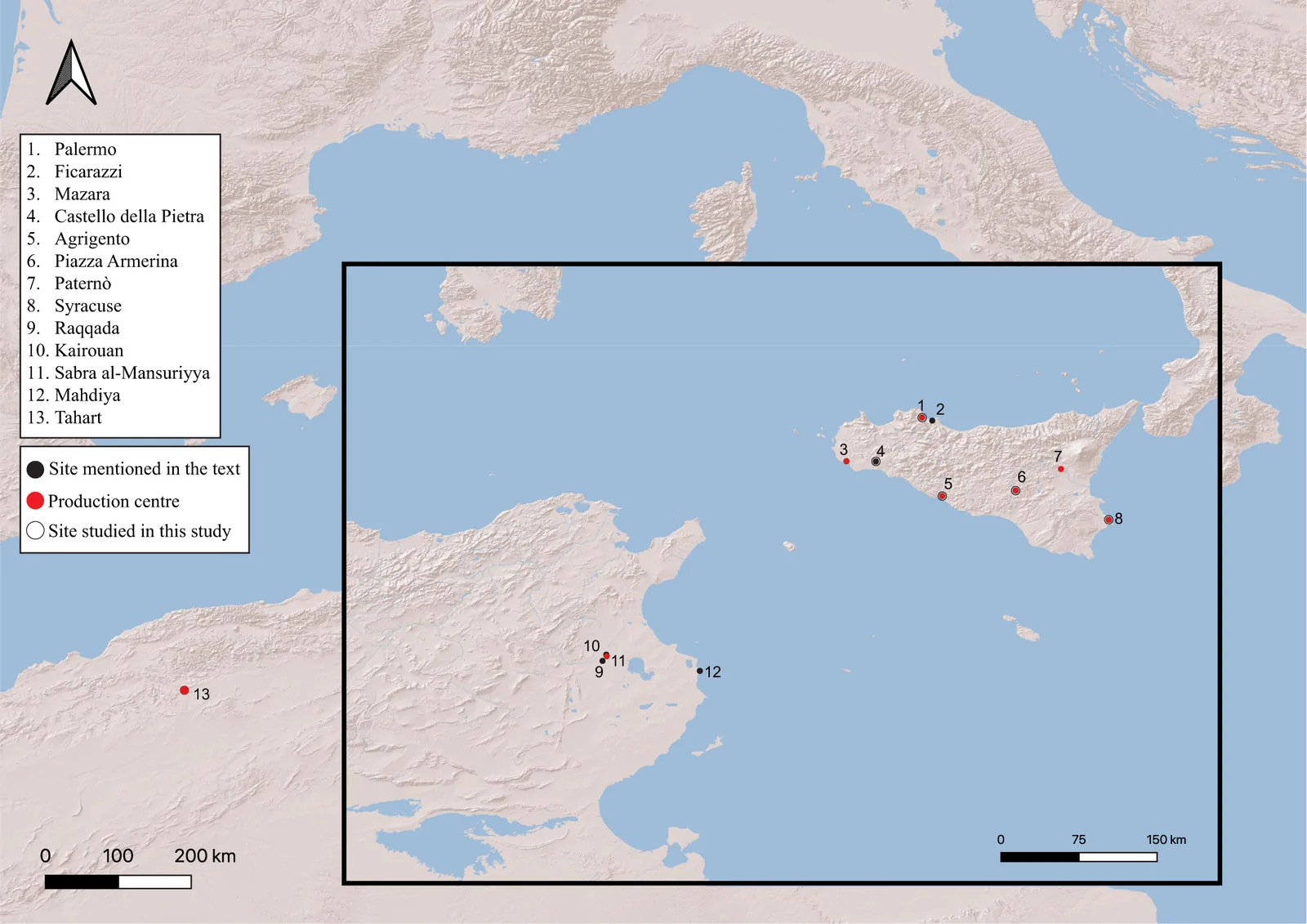
Location of archaeological sites mentioned in the text

No kilns dated from the late 9th to the 11th centuries AD producing glaze ware have yet been excavated in Sicily. However, scholars have identified at least six production centres through the discovery of wasters and kiln rods as well detecting reoccurring petrographic groups. These production centres were located in the capital (Palermo), three large urban centres (Mazara, Agrigento, Syracuse), a small urban centre (Paternò) and a rural settlement (Piazza Armerina) (Fig. 1). Archaeometric research on Sicilian glazed wares have primarily focused on the determination of provenance and the mapping of trade, with occasional studies on manufacturing technology (Testolini 2018) or residue analysis (Bramoullé et al. 2017; Pisciotta–Garnier 2018; Drieu et al. 2021; Lundy et al. 2021). Thin-section petrography has been the most widely used method, with geochemistry employed less frequently. However, the level of descriptive detail and geological interpretation varies between studies, making cross-comparison of previously published data difficult. 

Glazed tablewares in the highly distinctive Palermitan fabric, made of the so-called ‘Ficarazzi clay’, have been intensively studied petrographically (Capelli–Cabella 2024; Giarrusso–Mulone 2014; Montana et al. 2011; Testolini 2018). Although no kiln nor workshop dated to the 9th − 11th century has been discovered, thin section petrography confirmed that the Ficarazzi clay was employed to produce the full spectrum of ceramic wares of this period (e.g. Alaimo et al. 1999; Capelli–Cabella 2024; Giarrusso–Mulone 2014; Testolini 2018). To date, only one geochemical study has been carried out on a selection of glazed tablewares and amphorae sherds produced in Palermo (Waksman 2024). Its source, sedimentary alluvial clay deposit from the Lower Pleistocene, has been identified approximately 1 km east of the medieval city (Alaimo et al. 1999; Montana et al. 2011), in coastal areas extending from Acqua dei Corsari to Ficarazzi, near the mouth of the Eleuterio River, and extending to Solanto (Fig. 1). The grey-blue clay has been used since at least the Punic period (Giarrusso–Mulone 2014; Alaimo et al. 1998, 1999), up to the early 20th century (Alaimo et al. 1999: 49–50; Testolini 2018), as suggested by compositional analysis and ethnographic evidence (Alaimo et al. 1999: 49–50).

Studies on other Sicilian glazed ware production are more limited. At Mazara del Vallo, a local production centre has been known since the 1990s, following the discovery of a *bottega del vasaio* (potter’s workshop). Initially dated to the late 10th − 11th century, the workshop has been recently ascribed to the 11th century (Molinari–Meo 2021). Petrographic analyses were carried out on several glazed samples but distinguishing between Mazaran material and ceramics from North African proved difficult, as both are characterised by a calcareous fabric with a dominant presence of quartz inclusions (Capelli et al. 2021). Nonetheless, possible examples of Mazaran glazed ware ceramics have been identified outside Mazara itself, such as at Castello della Pietra, from where a selection of samples have been included in the present study (Capelli et al. 2023).

At Agrigento, evidence of glazed ware production, possibly dating back to the Islamic period, has been recently suggested by Rizzo et al. (2021: 438). Local production is primarily attested by the discovery of kilns and wasters in the artisanal district of *Fornaci S. Lucia*, where the pottery has been dated to the 12th century, probably the first half of the century. Most studies have focussed on these later typologies, and the local fabric is described as having dominant quartz as well as the presence of calcite and mica inclusions (e.g. Alaimo–Giarrusso 2007; Cuomo di Caprio 1992, 1995; Orecchioni 2021).

The existence of local ceramic production at Piazza Armerina has been hypothesised since the 1980s (Soustiel 1985: 212; Alaimo et al. 2010); Alfano’s (2018, 2019) and Bonanno–Canzonieri’s (2020) studies confirm that glazed tablewares were made between the late 10th − 11th centuries, as further supported by the discovery of kiln bars. In thin section, ceramics from Piazza Armerina are characterised by the presence of inclusions of monocrystalline quartz, feldspar and mica in a calcareous fabric (Alfano et al. 2019).

In eastern Sicily, traces of local glazed ware manufacture have been identified at two sites: Syracuse and Paternò. At Syracuse, evidence for local production near the Temple of Apollo comes from large quantity of wasters recovered during excavations in the early twentieth century. These indicate the manufacture of glazed wares between the late 10th and the first half of the 11th century (Fiorilla 2009). Petrographic and SEM-EDS analyses of five samples (Ben Amara et al. 2009) show that the ceramic fabric is characterised by a red clay matrix containing carbonate inclusions and volcanic rock fragments. More recently, evidence for local glazed ware manufacture has also been identified at Paternò, where the ceramic fabric is characterised by a quartz-rich composition with rarer inclusions of plagioclase, pyroxene, and amphibole (Messina et al. 2018).

In North Africa, the presence of kilns and/or wasters has been identified at three sites, all of which are possible centres of ceramic production. Several kilns have been excavated at the Fatimid-Zirid palatial site of Sabra al-Mansuriyya (Tunisia) on the outskirts of Kairouan. These provide evidence for the production of opaque white and turquoise glazed wares, as well as lustre wares, between the second half of the 10th and the 11th century. The products of this centre have been characterised via thin-section petrography and geochemistry (Capelli et al. 2011; Waksman et al. 2014). Another bar kiln has been found in the Fatimid-Zirid palace at Mahdia (Tunisia), but dated to the late 12th – 13th century and never fully studied (Louhichi 2000). Finally, kiln bars and wasters have also been found in the Rustamid capital of Tahart (Algeria), and some overfired sherds and kiln bars have been geochemically analysed (Djellid et al. 2023). However, macro-fabrics (Rossiter et al. 2012; Sacco 2024) and scientific studies of Ifriqiyan glaze wares (Capelli – Cabella 2024; Waksman 2024; Occari et al. 2024, 2025; Salinas et al. 2020, 2022) suggest the existence of several other unlocated production centres operating in the region from the late 9th to the 11th century. The primary analytical techniques employed are petrography and SEM-EDS, with fewer examples of XRF and LA-ICP-MS analyses. In addition to research on ceramics excavated from Tunisia and Algeria (Ben Amara et al. 2001, 2005; Capelli 2020; Occari et al. 2024, 2025; Salinas et al. 2020, 2022, 2023, 2024), some work has been conducted on possible Ifriqiyan ceramics exported to Sicily (Alaimo–Giarrusso 2007: 421; Alfano et al. 2019; Capelli et al. 2021, 2023, 2024; Sacco 2024) and the Italian peninsula (Mannoni 1979; Berti–Tongiorgi 1981).

## Study materials and sampling strategy

The sampling strategy was guided by the macroscopic classification of fabrics, itself informed by the petrographic studies noted in Sect. 2. The selection aimed to include the full range of identified local and non-local pottery, with no fabric categories excluded. A total of 188 samples were chosen from six sites in Sicily (Fig. 1, S1). The majority (*n* = 181) of them, have been selected from the urban centres of Palermo, Syracuse, and Agrigento and the settlement at Piazza Armerina, which are known to have produced glazed wares in the Islamic period. Seven samples were also included from the rural hilltop site of Castello della Pietra where no evidence of ceramic production has been identified. The site was nevertheless selected because previous studies identified the presence of Mazara production among the assemblage (Capelli et al. 2023). Table 1 presents a summary overview of the archaeological sites, assemblages and selected samples.

**Table 1:** Overview of the archaeological assemblages and sampling strategies from each site, including excavation context, dating basis, sample selection criteria, and the representativeness of the analysed ceramics within the available assemblages

Site	Type of site	Archaeological excavation and DMS coordinates	Excavation context and sampled assemblage	Sampling strategy	Dating	Summary of sampled ceramics	References
Palermo	City with documented local glazed production	Gancia (38°06’59.044"N, 13°22’14.115"E)	Excavations conducted in 2000 by the Soprintendenza di Palermo under the scientific direction of F. Spatafora. Eight trenches were opened within the area traditionally identified with the Fatimid *Ḫāliṣa*. The assemblage derives from stratified contexts recovered during systematic excavations. The sampled ceramics derive from deposits associated with the urbanisation of the area.	From systematic study of the excavated assemblage: MNI (only glazed wares) 2008.	Late 9th–mid 11th century CE, based on stratigraphic context and associated archaeological evidence.	Local: YELL YELLPNT YELLPNT/Palermo yellow TRANSPARENT POLYCH POLYCH/graticcio POLYCH/splash GREEN GREENPNT GREENINC BROW OPA OPA/TDA TUR OVERGLAZED MOULD Imports: YELL YELLPNT YELLPNT/Raqqada yellow POLYCH POLYCH/splash OPA TUR INCISED	Ardizzone et al. 2014; Sacco 2004.
		Bonagia Palace (38°06’57.2"N, 13°22’08.8"E)	Excavated between December 1997 and February 1998 under the direction of C.A. Di Stefano. A 15 × 20 m area in the palace garden was investigated through a grid of 5 × 5 m trenches. Although the complete excavation documentation is not available, the material derives from controlled archaeological excavations within stratified urban deposits.	From systematic study of the excavated assemblage: MNI (only glazed wares) 513.	Late 9th–mid 11th century CE, based on contextual attribution and associated archaeological materials.	Local: YELL YELLPNT YELLPNT/Palermo yellow POLYCH POLYCH/graticcio POLYCH/splash GREEN OPA OPA/TDA Imports: YELLPNT POLYCH OPA	Sacco 2004.
		Piazza della Vittoria (38°06′45″N, 13°21′19″E)	Material recovered during early twentieth-century excavations conducted by A. Salinas and/or E. Gabrici. No detailed excavation records or stratigraphic information are available. The assemblage therefore represents a legacy collection.	targeted sample selection from a systematic study of the assemblage: 69 MNI of glazed wares.	Second half of the 11th century CE, based on typological comparison.	Local: YELLPNT YELLST BROW CUERDA Imports: TUR	Pezzini et al. 2024.
Piazza Armerina	Settlement with documented local glazed production	Villa del Casale– Southern Baths Area (37°21’50.0"N, 14°20’02.9"E)	Samples derive from Trench II, SU 1792, and Trench V, SU 5048. These contexts consist of circular pits containing production waste, including kiln bars, wasters and discarded ceramics associated with workshop activity producing plain wares and storage vessels. The contexts are securely stratified and interpreted as production-related deposits.	Selection of samples from a systematic study of the assemblages: SU 1792 (320 MNI) and SU 5048 (70 MNI).	Late 10th–first half of the 11th century CE, based on contextual attribution and associated archaeological materials.	POLYCH POLYCH/SPLASH OPA/TDA GREEN TUR BROW	Alfano 2019.
		Villa del Casale– “Gentili Excavations” (37°21’50.0"N, 14°20’02.9"E)	Legacy assemblage recovered during 1950s excavations. Detailed contextual and stratigraphic information is unavailable.	Targeted sample selection; no reliable quantification is possible.	Second half of the 10th–first half of the 11th century CE, based on typological comparison.	POLYCH	Gentili 1999; Randazzo et al. 2018.
Syracuse	City with documented local glazed production	Temple of Apollo (37°03’47.8"N, 15°17’34.0"E)	Large quantities of kiln waste and production debris recovered during early twentieth-century excavations in the area of the Temple of Apollo. No stratigraphic documentation available. The material is interpreted as deriving from pottery production activities.	Targeted sample selection; no reliable quantification is possible.	Late 10th–first half of the 11th century CE, based on typological comparison.	OPA/TDA	Ragona 1986: 35–36; Fiorilla 2009.
Castello della Pietra Hilltop	Rural site	Castello della Pietra Hilltop (37°39’51.0"N, 12°53’29.5"E)	Investigated in 1973–1974 by E. Tomasello through two limited excavation trenches. Excavation methods were not fully stratigraphic. Islamic-period material was recovered from a series of circular pits or cavities, probably reused as refuse dumps.	Partially systematic study with targeted sample selection. No systematic quantification has been undertaken.	Late 10th–11th century CE, based on typological comparison and contextual attribution.	POLYCH TUR/Boli OPA/TDA GREEN OPA/TDA	Ardizzone et al. 2012.
Agrigento	City with documented local glazed production	S. Lucia Kilns (37°18’33.0"N, 13°34’43.1"E)	Material recovered during discoveries made in the late 1950s and subsequent excavations in the 1960s. The assemblage derives from a production area associated with pottery kilns, but detailed excavation records and stratigraphic information are unavailable.	Selected samples from the available material; no systematic quantification has been undertaken.	First half of the 12th century CE, based on typological comparison.	POLYCH GREENBROWN	Fiorilla 1995; Rizzo et al. 2021.

**Fig. 2:**
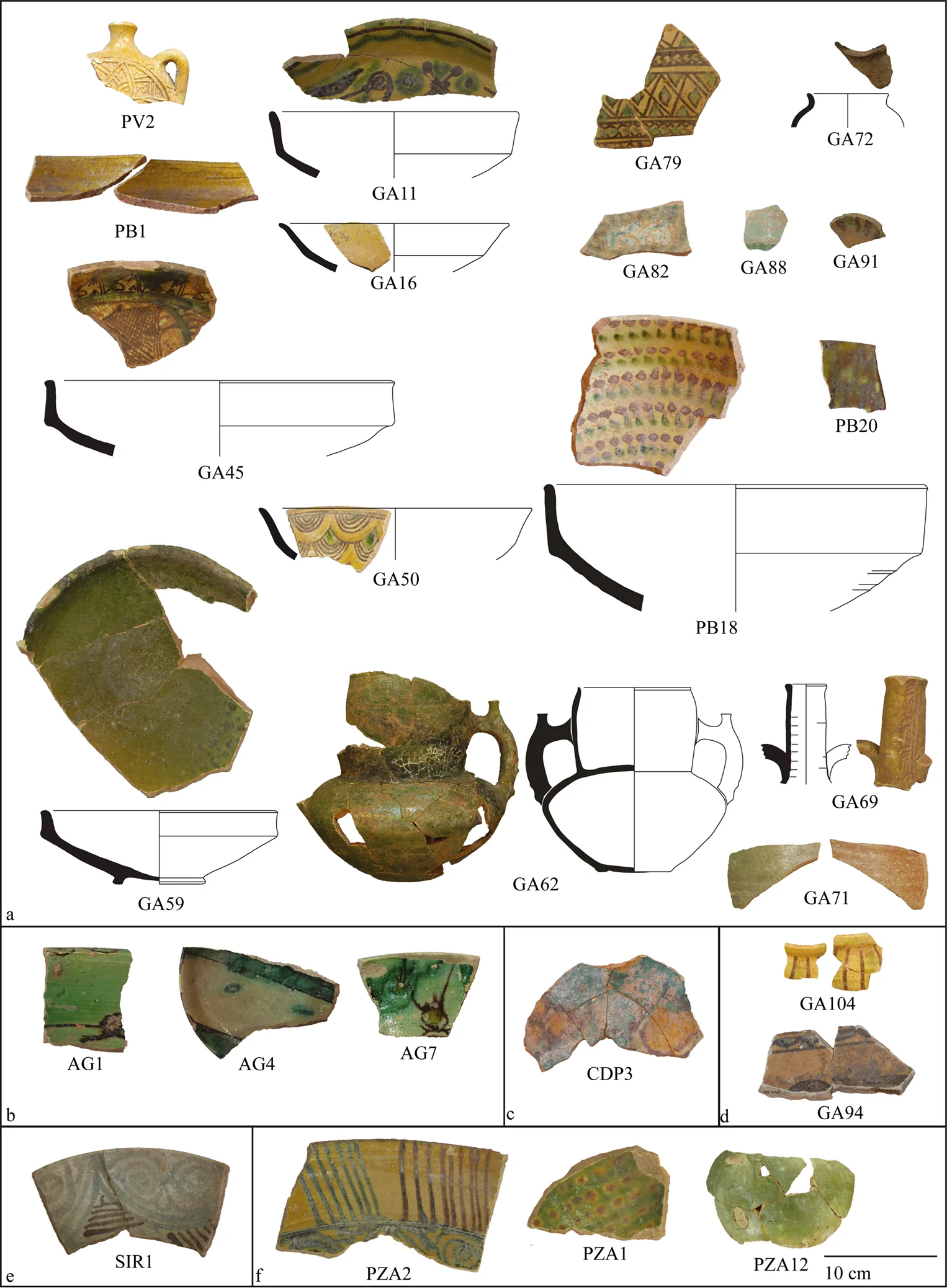
Images of the Islamic glazed ware ceramic samples analysed in this paper. **a**. Palermitan production; **b**. Agrigento production; **c**. Mazara production; d. Ifriqiyan production from Palermitan assemblages; **e**. Syracuse production; f. Piazza Armerina production (photographs by the author)

Samples were chosen to represent, as comprehensively as possible, the range of shapes, typologies, and macroscopic fabric groups ascribed to the five Sicilian production centres studied here (Table 2; Fig. 2). In the case of Palermo, for which a broader chronological range was available (late ninth–eleventh century), chronology was also taken into consideration during sample selection. Although no production waste or kiln rods could be sampled, as these materials were not accessible at the time of the study, previously published analytical data was included for comparative purposes. 

**Table 2:** Summary of known glazed typologies identified in the literature and refined by the authors

		Palermo	Mazara	Piazza Armerina	Syracuse	Agrigento	Ifriqiya
Transparent	Uncoloured Polychrome	late 9th -late 11th	late 10th -11th	late 10th -early 11th		early 12th ?	late 9th -late 11th
	Graticcio ware	mid 10th -early 11th					mid 10th -early 11th ?
	Splashed decoration	late 10th -early 11th		late 10th -early 11th			late 10th -early 11th ?
	Monochrome Yellow	mid 10th -early 11th					
	Yellow Polychrome	late 9th -early 10th					late 9th -early 11th
	Giallo di Palermo/jaune de Raqqada	late 9th -early 10th					late 9th -early 10th
	Monochrome Green	late 9th -late 11th	Late 10th -11th	late 10th -early 11th			late 9th -late 11th
	Green yellow cartoushe						late 9th -early 10th
	Green with brown decoration	late 10th -12th				early 12th ?	late 10th -12th ?
	Monochrome brown	mid 10th -early 11th		late 10th -early 11th			
Semi**-**Opaque	Whitish	late 9th -early 11th					late 9th -early 11th ?
	D’Angelo type	late 10th -11th	Late 10th -11th	late 10th -early 11th	late 10th -early 11th		late 10th -11th
Opaque	White	late 10th -11th					late 9th -early 10th and late 10th -11th
	Yellow dots	late 11th	late 11th ?				late 11th
	Green/Turquoise	late 10th -11th		late 10th -11th			late 10th -11th
	Luster						late 10th -11th

Of the 188 samples, 143 (S1) were selected from Palermo, primarily based on the systematic classification of Sacco (2024), which was based on the macroscopic study of > 2500 glazed sherds, supported by complementary petrographic and geochemical data on a representative subset. Three main glaze types were macroscopically identified with several stylistic subgroups defined based on decorative features (Table 2): transparent, semi-opaque, and opaque (Fig. 2). Transparent glazes include uncoloured, yellow, green, and brown variants, sometimes with underglaze decoration. Semi-opaque glazes, typically linked to polychrome decoration, may result from weathering or technical factors such as low tin content, unmelted quartz, and/or bubbles (Sacco et al. in press). Opaque glazes, white, green, or turquoise, often feature overglaze decoration.

For Palermo, sample selection focused primarily on securely dated contexts, although some undated pieces were included to ensure representation of the full typological and chronological range observed documented within its well-stratified assemblages. A total of 135 samples came from Gancia (*n* = 105) and Bonagia Palace (*n* = 30), both located in the Kalsa district, the area of the Fatimid-founded Madina Halisa (Table 1; Bagnera 2013; Sacco 2024: 31–33). The full range of the likely local glaze types was sampled (*n* = 119), taking into account their long chronological span (late 9th to 11th c.). A total of 24 samples interpreted as imports were also selected as well. This was complemented by eight additional sherds from Piazza della Vittoria, likely dating to the mid or late 11th century, included because certain glaze types were not represented, or were poorly represented, in the other two sites, likely due to the slightly later chronology of the Piazza della Vittoria contexts.

At the three other Sicilian production centres here considered (Table 1), glazed production began from the late 10th century, and exhibits a less diverse typological and stylistic range than the Palermitan and Ifriqiyan assemblages (Table 2). Unlike Palermo, where production spans from the late 9th to the 11th century, the well-stratified assemblage from Piazza Armerina is confined to a shorter chronological range (late 10th – first half of the 11th century). The selection of samples in this case was based on the systematic study of Alfano (2019). Sixteen samples were chosen to represent the variety of typologies produced at the site, which consisted of monochrome green and brown wares, polychrome wares with splashed or chess decoration, the so-called ‘D’Angelo type’ (TDA, a polychrome ware with a green and brown decoration traced with the same thickness) and possible green/turquoise ware. Based on previous studies, these samples are considered representative of the full range of locally produced glazed ceramics. For the remaining sites, the sample selection was constrained by the absence of stratigraphic information and, in some cases, by the lack of systematic studies. Twelve sherds dating to the late 10th first half of the 11th century were selected from the excavations at Syracuse, currently stored in the Caltagirone Museum. In the absence of stratigraphic information and systematic studies, selection was based on the available material and aimed to compare it with known typologies, all of which correspond to TDA ware. Ten sherds were selected from Agrigento, with a probable chronological attribution to the first half of the 12th century. Given the lack of systematic studies and later dating of the contexts compared to the others analysed in this study, selection focused exclusively on characterising the local fabric. The sherds selected are all polychrome wares, with the exception of two samples, which are green glazed with brown painted decoration under the glaze. Finally, seven sherds were sampled from the rural hilltop site of Castello della Pietra, five of which had previously been identified as originating from Mazara (Capelli et al. 2023). For this site, only recognisable sherds were selected, mainly represented by TDA and *boli gialli* type, specifically targeting those previously identified as Mazaran production.

## Methodology

The paste of glazed ware sherds was characterised and classified using a combination of macroscopic fabric, thin-section petrography and instrumental geochemistry. Macroscopic fabric analysis was conducted on a fresh break with a 60–100× portable microscope, following Sacco (2024), adapted from established methods (Orton et al. 1993; Rice 1987: 274–306). The sherds were splits into macro-fabrics based on the colour of their matrix as well as texture, hardness, fracture, inclusion and void characteristics. The resulting macro-fabrics were used to select samples for petrographic and geochemical analysis.

A total of 188 sherds were mounted in resin blocks and prepared as standard 30 μm thin sections (Quinn 2022: 23–36) at the Wolfson Archaeological Science Laboratories (UCL). Petrographic analysis was conducted using a LEICA DM EP Polarising microscope at magnifications of 40–200x. Samples were visually grouped into petrographic fabrics in terms of their main inclusions and the nature of their clay matrices, then described in detail following a modification of the Whitbread system (1995, 2016), devised by Quinn (2022).

The bulk geochemical composition of 150 glazed sherds was recorded via instrumental neutron activation analysis (INAA) at the University of Missouri Research Reactor (MURR). Samples measuring approximately 1 cm^2^ were prepared according to in-house protocols (Glascock 1992 and https://archaeometry.missouri.edu/drilling.html) by clipping off the glaze and removing the uppermost part of the fabric with a tungsten-carbide drill. The samples were cleaned in deionized water and dried before crushing and grinding. They were then exposed to two irradiations and three gamma counts (Glascock 1992, 2019; Glascock–Neff 2003; Neff 2000), resulting in the qualification of a total of 33 elements (Al, As, Ba, Ca, Ce, Co, Cr, Cs, Dy, Eu, Fe, Hf, K, La, Lu, Mn, Na, Nd, Ni, Rb, Sb, Sc, Sm, Sr, Ta, Tb, Ti, Th, U, V, Yb, Zn, and Zr). Approximately 150 mg of powder was placed in a high-density polyethylene vial for a short (5-second) irradiation, after which gamma-ray emissions were recorded. For the long irradiation (24 h), 200 mg aliquots were sealed in high-purity quartz vials to target isotopes with longer half-lives. Several NIST certified standard reference materials SRM-1633b (Flyash), SRM-688 (Basalt Rock), SRM-278 (Obsidian Rock) and the New Ohio Red Clay (Glascock 1992) were analysed alongside the archaeological unknowns for calibration and quality control purposes (S2 for accuracy and precision of the standards).

The resulting multivariate geochemical dataset was explored statistically using principal component analysis (PCA) and hierarchical cluster analysis (HCA) to identify compositional groupings. The resulting geochemical patterning was then compared with the macroscopic and petrographic fabric classification, as well as the find spot and typology of the sherds, in order to identify compositional signatures and assess their likely provenance. Petrographic data was available for all sites and therefore provided the principal framework for inter-site comparison, whereas geochemical comparisons were limited by the availability of published reference datasets, which are restricted to Palermo and Ifriqiya. Comparisons with these reference groups rely on previously published compositional data generated using different analytical techniques, including XRF and LA-ICP-MS. Although inter-laboratory comparability issues, particularly for trace elements, cannot be fully excluded, previous studies have demonstrated a generally good correspondence between INAA, LA-ICP-MS, and XRF datasets (Dussibieu et al. 2007; Little et al. 2004; Speakman et al. 2007; Stoner 2016). No specific correction factors were applied, as data on common certified reference materials was not available for all the published datasets included. Nevertheless, an important validation of the comparability comes from the overlap between the compositional groups identified in this study and in previous works.

No raw material sampling or analysis has been undertaken in the present study. Nevertheless, comparisons have been made between the geological characteristics of the ceramic fabrics and the nature of the bedrock and superficial sediments occurring near each site, as well as with published compositional data.

Because the assemblage consists entirely of glazed ceramics, the potential contribution of glaze-derived elements to the bulk ceramic composition was considered. To assess possible glaze–body interaction and evaluate the risk of elemental diffusion, glazed surfaces and ceramic bodies were examined by SEM–EDS. A detailed characterisation of glaze compositions and technological aspects lies beyond the scope of the present study and will be presented separately. Here, glaze-related observations are discussed only insofar as they are relevant for assessing their potential influence on the geochemical characterisation of the ceramic fabrics.

## Results

### Macroscopic fabrics

**Fig. 3:**
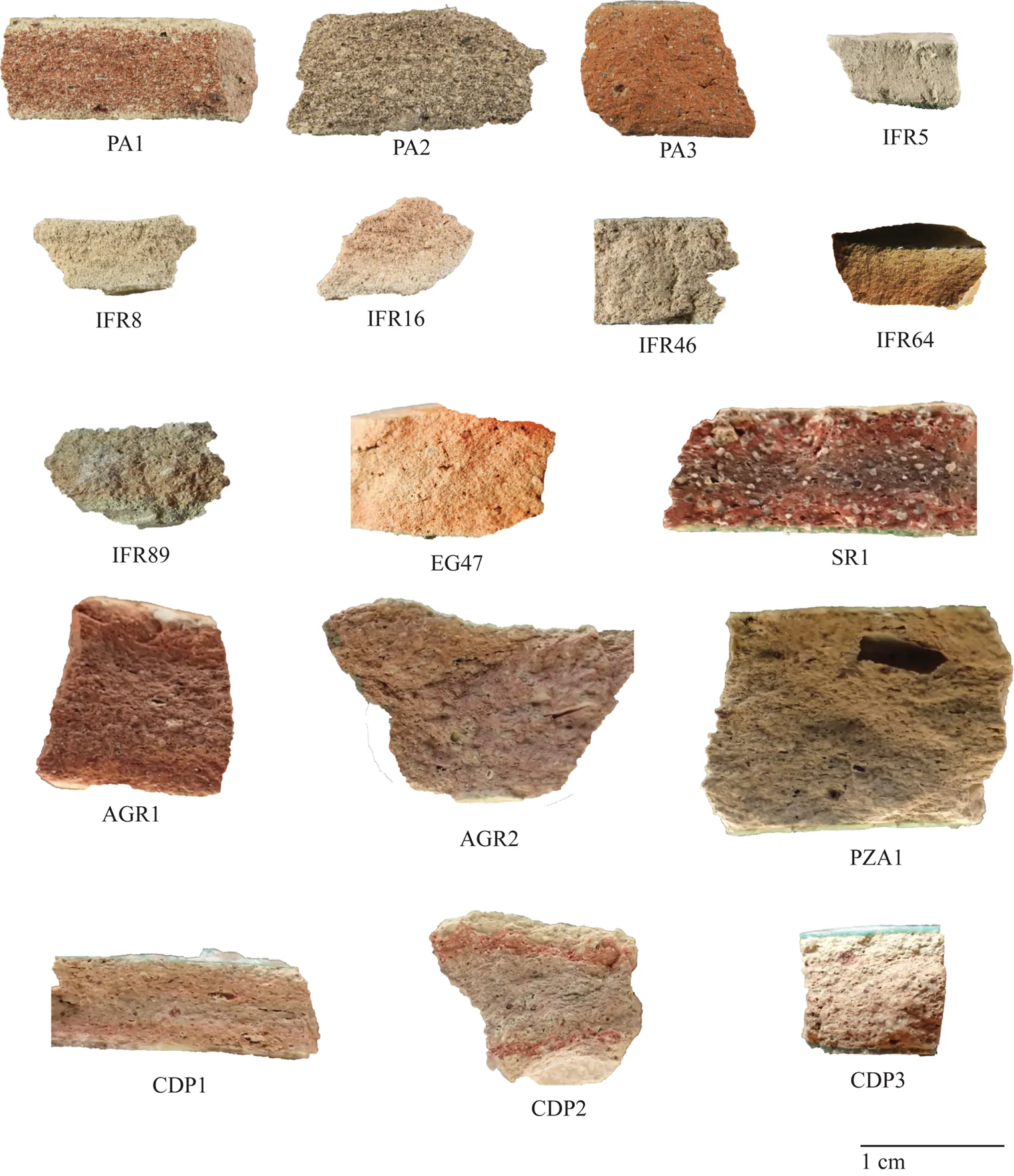
Representative photographs of the 15 macroscopic fabric groups identified in the studied assemblage (photographs by the author)

A total of 15 macroscopic fabrics, described in detail in the S3, were identified (Fig. 3). Ten fabrics were recorded from three sites located in Palermo (Gancia, Palazzo Bonagia, and Piazza della Vittoria) and published in Sacco (2024), from which the current numbering system is derived. Among these, PA1, PA2 and PA3 are interpreted as locally produced. They differ primarily in the colour of their ceramic matrices, likely the result of different firing conditions. A characteristic ‘salt-white’ surface (Ownby–Griffith 2009) was observed on nearly all the sherds in these fabrics, suggesting a common production technique. According to the provenance identifications proposed by Sacco (2024), six fabrics are likely to be imports from Ifriqiya (IFR5, IFR8, IFR16, IFR46, IFR64, and IFR89), while EG47 may represent an Egyptian import.

Additional macroscopic fabric categories were identified within the sampled sherds. Two possible local fabrics (AGR1 and AGR2) were distinguished at Agrigento; the differences between them are subtle and most likely attributable to variation in firing atmosphere or temperature rather than to differences in raw materials or production centres. The Syracuse assemblage yielded only one probable local fabric (SR1), while the fabric attribution of three additional sherds (SIR10/VSC053, SIR11, SIR12) remains macroscopically uncertain. At Piazza Armerina, one fabric (PZAR1) is considered local based on previous studies (Alfano et al. 2019). Three main macroscopic fabrics were discerned at the site of Castello della Pietra: CDPT1, CDPT2, and CDPT3. These share similar inclusions, with the primary differences related to the colour of the fabric that likely reflects different firing conditions rather than separate recipes. Notably, the fabrics, especially the lighter-coloured variant of CDPT3, exhibit similarities with IFR8 and IFR16, differing mainly in the more abundant presence of white, holed inclusions. However, we cannot entirely exclude the possibility that some of the sherds classified as CDPT3 may belong to the same fabric group as IFR8 or IFR16. 

### Thin section petrography

**Fig. 4:**
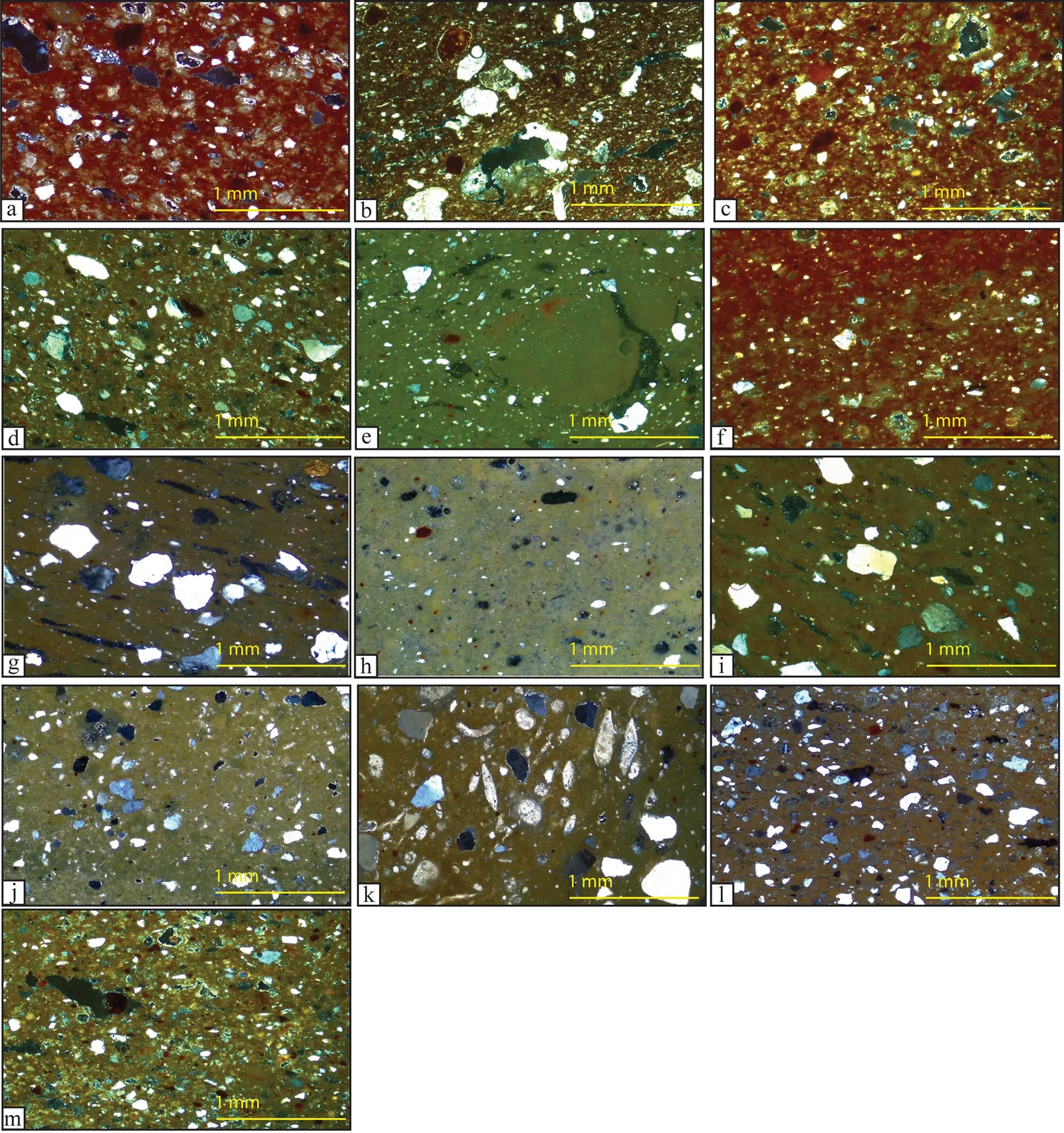
Thin section photomicrographs of the fabrics identified: (**a**) Fabric 1 (PB30); (**b**) Fabric 2 (SIR4); (**c**) Fabric 3 (SIR10); (**d**) Fabric 4 (PZA5); (**e**) Fabric 5 (PZA6); (**f**) Fabric 6 (AG2); (**g**) Fabric 7 (CDP4) (showing amphibole); (**h**) Fabric 8 (GA105); (i) Fabric 9.1 (PV6); (**j**) Fabric 9.2 (GA94); **k**. Fabric 10 (PV7); **l**. Fabric 11 (GA94); **m**. Fabric 12 (GA100). All images taken in crossed polars (XP). Image width = 3.2 mm

A total of 179 sherds were classified into 12 petrographic fabrics in thin section (Fig. 4), one of which was further subdivided into sub-fabrics (for the full description see S4). Fabric 12 (Fig. 4m) is petrographically distinct and is interpreted as an Egyptian production and will not be discussed in this paper.

All analysed ceramic samples were highly fired, as suggested by the low optical activity of their clay matrices (Quinn 2022: 114, 269, 273). This is further supported by the alteration of the calcite in limestone and shell inclusions, as well as of other inclusions such as glauconite, amphibole and mafic material. The high firing posed some difficulties for the grouping of the sherds in thin section.

The most abundant fabric is Fabric 1 (Ficarazzi Fabric, Fig. 4a, *n* = 114), a non-calcareous fabric characterised by a predominant monocrystalline quartz and frequent micritic limestone, which exhibits different degrees of calcite degradation. Other inclusions include common ferruginous nodules, occasional chert, rare bioclasts, and very rare to absent glauconite, plagioclase feldspar, clinopyroxene, muscovite and biotite mica. The matrix is, with few exceptions, heterogeneous, and ranges in colour from red to dark brown. Many samples feature light coloured halos around degraded calcareous inclusions, many of which are represented by voids. In the present dataset it has been identified mainly in the Palermitan sites (Gancia, Bonagia Palace and Piazza della Vittoria), and perhaps in Syracuse and Piazza Armerina.

The next two fabrics identified, Fabric 2 (Calcite and Altered Mafic Tempered Fabric, Fig. 4b, *n* = 8) and Fabric 3 (Calcite and Glauconite Fabric, Fig. 4c, *n* = 3), contain rare quartz and were dominated by subrounded and subangular limestone inclusions, which were likely added as temper. Both are non-calcareous: Fabric 2 contains mafic weathered material, possibly iddingsite or serpentinite, while Fabric 3 is differentiated by its common glauconite. Other inclusions identified in Fabric 2 include ferruginous nodules, chert, plagioclase feldspar, biotite and muscovite mica, altered igneous rock (such as basalt), amphibole, and clinopyroxene. Fabric 3 is finer grained and contains only biotite and muscovite mica, chert, and ferruginous nodules as inclusions. This may suggest that the clay was refined. They have both been identified in Syracuse corpus.

Few biotite-rich fabrics, grouped under Fabric 4 (Biotite-rich Calcareous Sandy Quartz Fabric, Fig. 4d, *n* = 10), contain sand-sized quartz and subordinate microcline and plagioclase feldspar, muscovite, chert and common ferruginous nodules. Fabric 5 (Calcareous Silty Quartz Fabric, Fig.4e, *n* = 4) is distinguished from Fabric 4 by its more silty grain size and a lower proportion of biotite. In both cases the presence of regions of clay devoid in inclusions suggest that quartz was added as temper (Quinn 2022: 230–231). Fabrics 4 and 5 occurred only in Piazza Armerina.

Within the Agrigento assemblage a non-calcareous matrix dominated by monocrystalline quartz, frequent micritic limestone fragments, few bioclasts, and ferruginous nodules (Fabric 6, Biotite Rich Non-calcareous Silty Quartz Fabric, Fig. 4f, *n* = 10) has been identified. It is distinguished by the presence of few to common biotite mica grains and some subordinate inclusions, including amphibole and microcline feldspar. The main variation within this fabric is the presence or absence of olive green-coloured patches, which are compatible with the degradation of calcite and its reaction to the salt mixed with the clay. Finally, the clay matrices of some samples are optically slightly active, indicating that they did not completely vitrify.

A distinct bimodal distribution of quartz inclusions characterise Fabric 7 (Calcareous to Moderately Calcareous Fabric with Bimodal Quartz Inclusions, Fig. 4g, *n* = 5), documented in Castello della Pietra, which also displays a coarse fraction ranging from fine to medium sand. It is also characterised by occasional biotite mica and microcline feldspar, elements which distinguish it from the fabrics below. Fabrics 8 to 11 have been identified in Palermitan assemblages and one from Castello della Pietra (Fabric 9.1). They all share a generally similar mineralogical composition, characterised mainly by rounded, subrounded to angular monocrystalline quartz grains and ferruginous nodules. However, variations in the amount, distribution, and size of inclusions, as well as in the subordinate mineral content, permit subdivision into four main petrographic fabrics and one sub-fabric. Fabric 8 (Highly Calcareous Fabric with Sparse Quartz, Fig.4h, *n* = 2) is characterised by a low inclusion content and a highly calcareous matrix, clearly distinguished by the limited quantity and sparse distribution of inclusions. Fabric 9 (Moderately Calcareous Sandy Quartz Fabric, Fig. 4i-j, *n* = 11) exhibits quartz sand giving it a strongly to moderately bimodal grain size distribution and has been divided into two subfabrics. Subfabric 1 has a distinctly bimodal distribution, with the coarse fraction ranging from fine to medium sand; Subfabric 2 exhibits weaker bimodality, with the coarse fraction ranging from very fine to fine sand. A further distinguishing feature is the presence of elongate voids in Subfabric 1, which are absent or extremely rare in Subfabric 2. In both subfabrics, subordinate inclusions such as rare to absent plagioclase and biotite mica, amphibole, and chert have been identified. Fabric 10 (Calcite and Quartz-Temper Calcareous Fabric, Fig. 4k, *n* = 1) is similar to Fabric 9 but stands out due to the presence of frequent micritic limestone fragments and bioclasts. This fabric is represented by a single sample and may share the same clay source as Fabric 9, though the observed differences likely reflect a distinct technological choice during production.

Fabric 11 (Slightly Calcareous Fine Sandy Quartz fabric, Fig. 4l, *n* = 9) is a fine sandy quartz fabric that shares broad similarities with Fabrics 8 and 9, particularly in terms of the types of inclusions present. However, it can be distinguished by its better-sorted quartz grains and the more discrete, evenly spaced distribution of inclusions throughout the matrix. Despite these distinguishing features, in practice it was not always straightforward to differentiate Fabric 11 from Fabric 9, due to overlapping inclusion sizes and textures in some samples.

All the samples, except for Fabrics 4, 5 and 8, feature a light-coloured vitrified layer beneath the glaze, that is clearly distinguishable from the rest of the body. This varies in thickness (0.25–1.5 mm) and in the case of open forms, it generally appears on both sides of the vessel. It contains the same inclusions as those found in the ceramic matrix and has an irregular merging boundary with the body below, which differentiates it from slip coatings. This suggests that this zone does not represent a separate decorative layer. Previous research has interpreted such layers as being formed by the migration of deliberately added salt during the drying process and is referred to as a ‘salt white surface’. The salt locally fluxes the clay minerals, resulting in surface vitrification (Ownby–Griffith 2009: 232). This technique has been employed by Sicilian and North African artisans since at least the Punic period (Peacock 1984), and it is still in use today in Tunisia (Dufournier 1982; Ownby–Griffith 2009: 230). 

### Geochemistry

The concentrations of 33 major (Fe, Al, Ca), minor (K, Mn, Na, Ti) and trace elements (As, La, Lu, Nd, Sm, U, Yb, Ce, Co, Cr, Cs, Eu, Hf, Ni, Rb, Sb, Sc, Sr, Ta, Tb, Th, Zn, Zr, Ba, Dy, V) in the 150 sherds analysed by INAA (S5) was explored statistically via principal component analysis (PCA) and hierarchical cluster analysis (HCA) to identify geochemical patterning. The dataset was log-ratio transformed to convert compositional values into logarithms of ratios between components (or relative to a reference), thereby addressing the constant-sum constraint inherent in compositional data and reducing the dominance of highly abundant elements in multivariate analyses such as PCA and hierarchical clustering (Aitchison 1986).

Given that the assemblage consists entirely of glazed ceramics, particular attention was paid to elements that could potentially reflect glaze-derived contributions rather than the composition of the ceramic fabrics. SEM–EDS microstructural analysis of 120 glazed samples, (see Occari et al. 2025 for methodology) indicates that most specimens exhibit either a very thin glaze–body interaction zone or no clearly observable interaction layer. The glazes are characterised by consistently high lead contents (40–65 wt% PbO), accompanied by variable amounts of alkali fluxes (Na_2_O+K_2_O, 0.05-6 wt%). Colouring agents conform to established technological practices, with CuO producing green colours, iron oxides yellow/light brown tones, and manganese oxides brown/black colours, often in combination with iron. Antimony oxide occurs only sporadically and is associated with a specific pottery type (*boli gialli*), and identified as an opacifier in a single sherd. Opacification was achieved through the addition of SnO₂ and/or the incorporation of unmelted quartz particles.

Prior to multivariate analysis, the dataset was evaluated through two complementary screening procedures: analytical quality control and assessment of elements potentially affected by glaze-related contributions. Elements presenting missing values, concentrations below the limits of detection, poor accuracy, precision or showing abnormally high variation, potentially indicative of post-depositional alteration, were first excluded to minimise analytical error (Buxeda y Garrigós 1999; Buxeda y Garrigós et al. 2001; Freestone et al. 1985; Quinn 2022; Schneider 2016). Based on the measurement of certified reference materials, the relative error for the element uranium was calculated at 14.8%, and for sodium at 12.38% (S2). Given these high levels of uncertainty, both elements were excluded from the dataset. Nickel was also removed as it contains zero values for several samples (S5).

Among the remaining elements quantified by INAA, Sb, Mn, K and Fe were also evaluated as potential contributors to the bulk compositional dataset because these elements may be associated with glaze materials or glaze–body interactions during firing (Molera et al. 2001). Cobalt, another common glaze colourant, is absent from the assemblage and therefore does not contribute to compositional variability. For this dataset, the possibility that glaze contamination influenced the bulk chemical composition is considered unlikely for several reasons. Firstly, the ceramics were subjected to a double-firing process, in which the body was bisque fired before glaze application, then subjected to a second firing step to form the glaze. As a consequence, the main compositional characteristics of the ceramic body were established prior to glazing. Secondly, the glaze-body interaction layer is absent or very thin so diffusion of elements from the glaze into the already fired body is likely to the have been minimal. Removal of the topmost layer of the fabric during preparation will nevertheless have ensured that possible contaminants were excluded from the INAA body data. Finally glaze recipes appear broadly comparable across the analysed samples, so should glaze-derived contamination have occurred, it would be expected to produce a relatively uniform modification of the ceramic bodies rather than the distinct compositional groups identified by PCA and HCA.

**Fig. 5:**
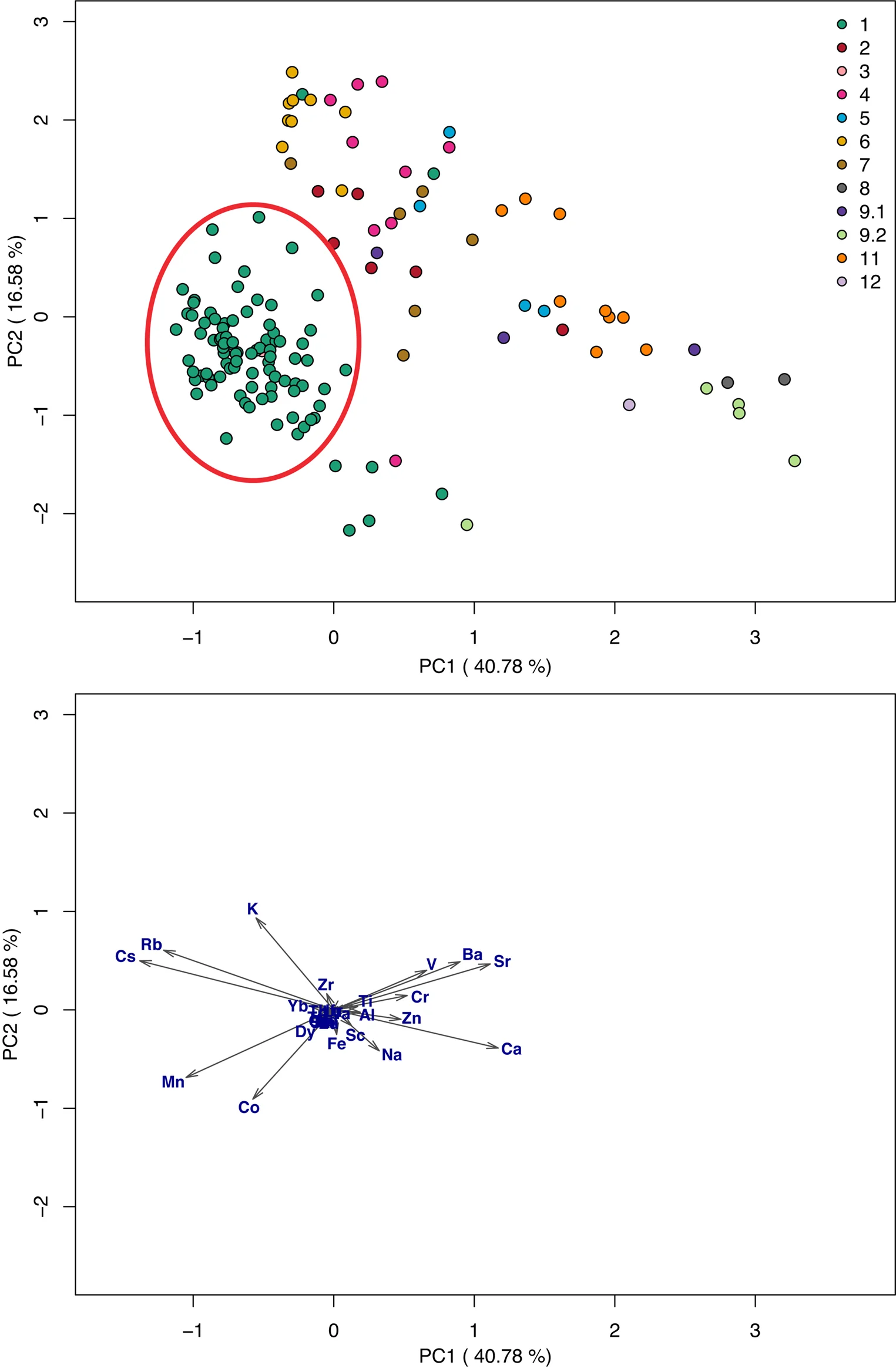
Principal component analysis score plot (PCA) of 149 ceramic samples based on 29 major, minor, and trace elements measured by INAA, showing the main geochemical patterning within the assemblage

The distribution of elements potentially associated with glaze contamination further supports this interpretation. Antimony occurs only in trace and sporadic amounts and is not considered analytically significant at the assemblage scale and was therefore excluded. As shown in Fig. 5, Fe does not exert a discernible influence on the multivariate structure of the dataset, while Mn variability is independent of the presence of manganese-bearing glazes. In particular, within the same geochemical groups, samples with Mn-coloured glazes and samples without Mn-bearing surface decoration exhibit comparable Mn concentrations, indicating that the presence of manganese in the glaze did not measurably affect the bulk composition of the ceramic bodies. Similarly, K concentrations do not show evidence of systematic glaze influence. Potassium contents in the glazes are relatively low (approximately 0.5–2.5 wt%), and the analytical samples were obtained by drilling into the ceramic body after mechanical removal of the glaze layer, substantially reducing the likelihood that residual glaze material affected the compositional dataset. Finally, the relatively dense fabric and low porosity of the ceramic bodies make significant migration of glaze components into the body during firing unlikely. Taken together, these observations indicate that glaze contamination did not significantly influence the bulk chemical composition or the resulting multivariate analyses.

Despite evidence in thin section for the degradation of calcite inclusions during and after firing in several samples, calcium was retained in the dataset because calcite temper had been added to some fabrics and was therefore important for the petrographic classification of the ceramics. Moreover, previous experimental studies (e.g. Schneider 2016: 167) suggest that secondary calcite does not significantly alter the original calcium content of ceramics unless extensive cracks or open pores are present for crystallization; as the samples analysed here show no significant cracking or large voids, and secondary calcite was identified in only one specimen (S4), its impact is likely minimal. Exploration of the dataset both with and without calcium reveals similar patterning, suggesting that the degradation of the calcite inclusions had little effect on the composition of the ceramics.

A principal component analysis scores plots based on the retained major, minor, and trace elements explains 57% of the total variance and reveals moderate separation among the samples (Fig. 5). A compact compositional group consisting primarily of Palermitan petrographic Fabric 1 samples is clearly visible on the left side of the plot and also includes the single analysed sample of Fabric 3. This group is characterised by elevated concentrations of several correlated trace elements, including Co, Cs, Mn, and Rb.

The remaining samples are more broadly dispersed across the plot, indicating greater geochemical variability. On the right side of the plot, two more dispersed compositional tendencies can be observed, associated with higher concentrations of Cr, V, Ba, Sr, and Zn. One is characterised by higher potassium concentrations and includes samples assigned to Fabrics 2, 4, 5, 6, and 7 (the Sicilian fabrics). The other is characterised by higher calcium concentrations and includes Fabrics 8, 9, and 11 (the North African fabrics). Although the samples belonging to these samples are more chemically variable than the main Palermitan group, they nevertheless display internal compositional consistency. The broader dispersion of these samples may partly reflect genuine compositional variability; however, it may also be influenced by the smaller sample populations available for these fabrics, which limit the ability to define more robust compositional groupings. Increasing the number of analysed samples in future studies will be necessary to further refine these groupings.

**Fig. 6:**
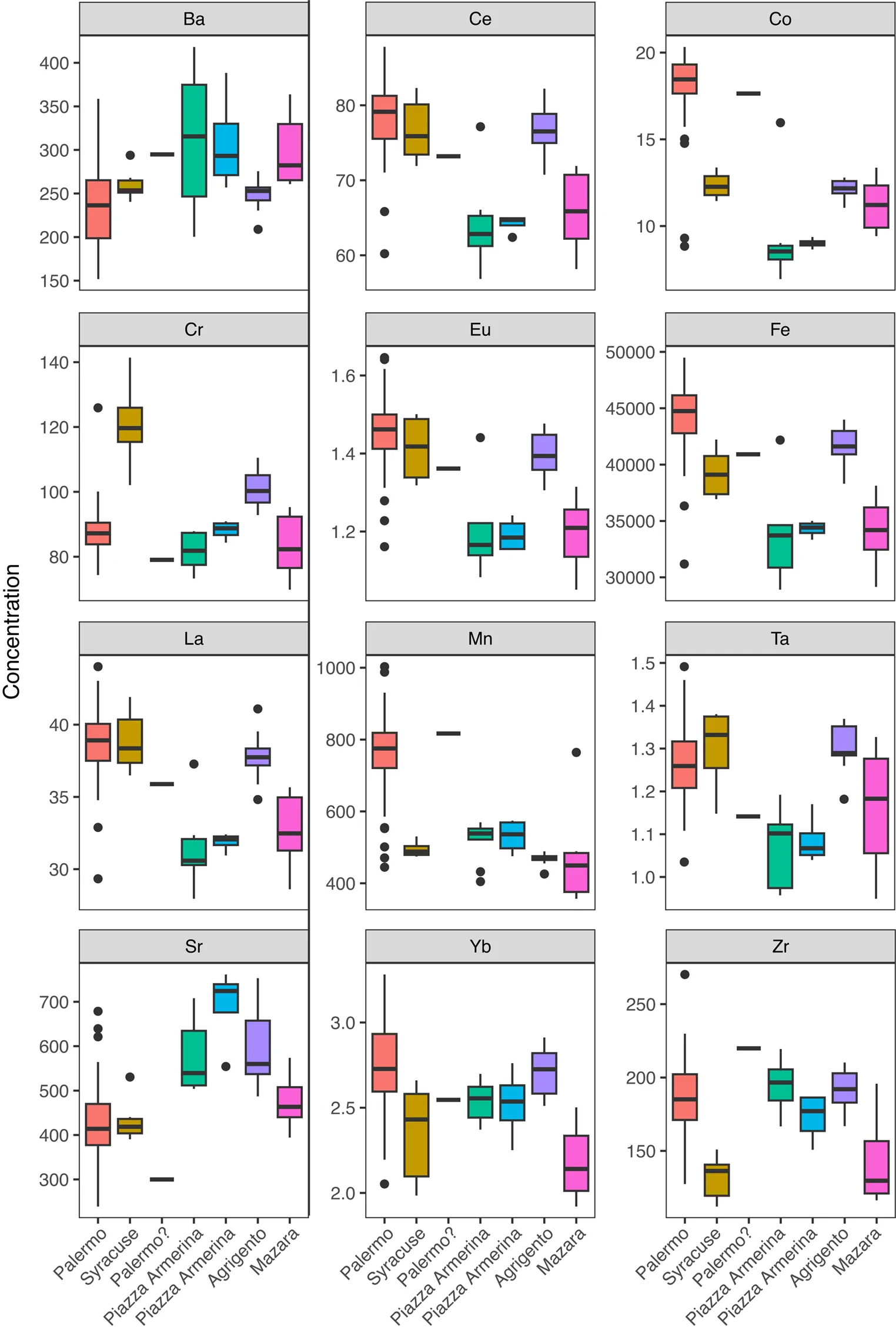
Box plots of the geochemical variability of selected major, minor and trace elements within the Sicilian medieval galzed ware ceramics classified in petrographic fabrics 1–7 in this study

Boxplots of the geochemical variability of the samples belong to the seven Sicilian-attributed petrographic fabrics (Fig. 6) reveal differences primarily in calcite and iron, while the minor and trace elements, which are often employed as provenance indicators (Mommsen 2001; Mommsen et al. 1991), reveal distinctions in terms of variability in the concentrations.

**Fig. 7:**
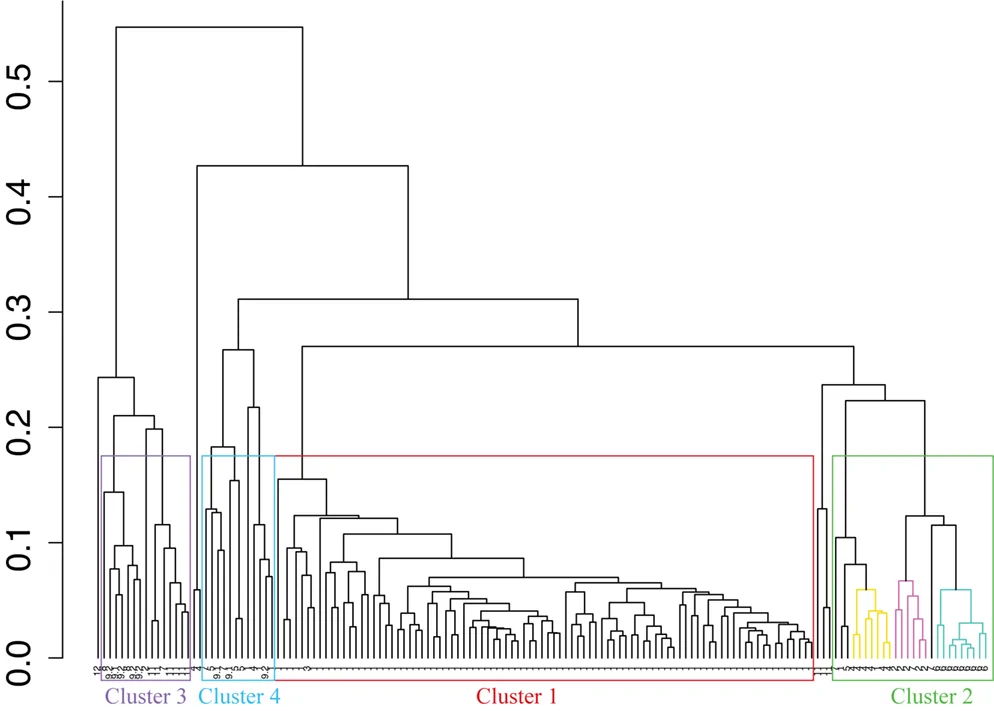
Hierarchical cluster analysis of trace and minor elements showing the four clusters identified, the three subgroups within Geochemical Cluster 2 and the outliers

Average-linkage hierarchical cluster analysis (HCA) was performed on the minor and trace elements only and the resulting dendrogram reveals four clusters, consisting of one dominant group (Geochemical Cluster 1) and three smaller groups (Geochemical Clusters 2–4). Additionally, six samples (VSC004, VSC023, VSC024, VSC025, VSC047, VSC048) fall outside these main clusters (Fig. 7).

Consistent with the patterns observed in the PCA, Geochemical Cluster 1 represents the most abundant compositional grouping (*n* = 89), including the majority of Fabric 1 samples and the only Fabric 3 sample analysed, encompassing therefore the majority of the analysed samples. Geochemical Cluster 2 (*n* = 26) includes specimens that correspond mainly to three petrographic fabrics (Fabrics 2, 4 and 6). Interestingly, this cluster contains finer-scale chemical patterning that corresponds with the petrographic subdivisions, as samples attributed to Fabric 2, 4 and 6 each form distinct sub-groups within the cluster.

Geochemical Cluster 3 (*n* = 15) comprises mainly samples from Fabrics 8, 9 and 11, which are suspected to be North African in origin. In contrast, Geochemical Cluster 4 (*n* = 12) includes samples belonging to different petrographic fabric groups. Their greater internal variability and weaker petrographic coherence may reflect overlaps in raw material procurement or problematic values. 

## Discussion: integrating macroscopic, petrographic, and geochemical data to characterise Islamic-period glazed wares in Sicily

The combined petrographic and geochemical classification of Islamic-period glazed ware sherds from Sicily in this study, confirms the existence of ceramics from several distinct production centres. The likely geographic origin of these production centres is inferred based on the combined macroscopic, petrographic and geochemical characterisation of ceramic fabrics and comparison with published archaeological and geological data. As no systematic survey of potential raw materials was undertaken, proposed links between fabrics and specific clay sources should be regarded as indicative rather than definitive.

**Fig. 8:**
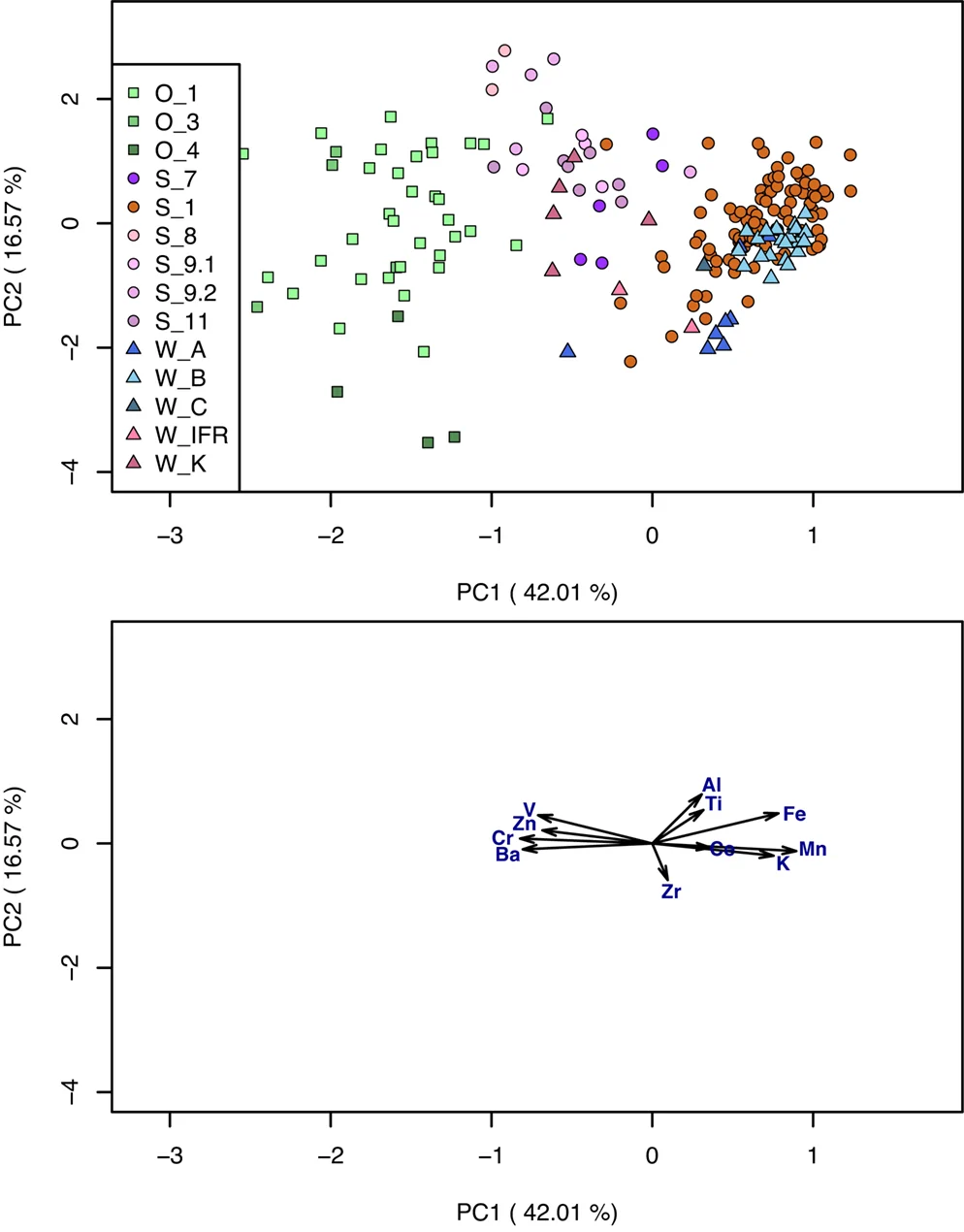
Multivariate statistical exploration of 149 sherds of glazed ware ceramics, based on 11 elements (Al, Ba, Ce, Cr, Fe, K, Mn, Ti, V, Zn, Zr) using centered log-ratio transformed data for the fabrics attributed to Palermo and Ifriqiya compared with the glazed pottery fabrics published by Waksman 2024 and Occari et al. 2025 (O = Occari et al. 2025; S = our dataset; W = Waksman 2024)

The clearest and most coherent group corresponds to wares likely produced in Palermo (Petrographic Fabric 1/Geochemical Cluster 1), found mainly in Palermo. The tight petrographic and geochemical composition of this set of samples suggests the use of a highly consistent paste recipe (Arnold 2000) and perhaps the exploitation of a restricted range of raw materials. Previous studies have linked comparable fabrics to the so-called ‘Ficarazzi’ clay, a grey-blue clay characterised by common quartz, fine calcite sand and foraminifera microfossils (e.g. Montana et al. 2011; Testolini 2018). Although potential biases arising from the comparison of datasets produced using different analytical methods and in different laboratories must be acknowledged, multivariate statistics conducted on the geochemical data from the present project, with the previous datasets of Waksman (2024) and Occari et al. (2025), show strong overlap between the Petrographic Fabric 1 sherds and previously analysed samples ascribed to Palermo, while clearly separating them from North African ceramics (Fig. 8). This also supports the general inter-comparability between the datasets collected with different analytical techniques. However, a compositional subgroup of late 9th – early 10th century sherds (Waksman 2024: 315–316) could not be recognised in the present dataset, most likely because only a limited number of securely dated specimens from this phase were available.

A small number of sherds in this study that are likely of Palermitan origin show chemical differences in certain elements, which may be attributable to technological variation or analytical inconsistencies. Similarly, samples VSC028 and VSC029, interpreted macroscopically and petrographically as Palermitan imports at Piazza Armerina, align chemically with Geochemical Cluster 2, suggesting either chemical overlap between Sicilian fabrics or a possible misattribution.

In other cases, petrographic and chemical analyses can be compared to assess relationships between fabrics. Petrographic Fabric 3, identified in Syracusan assemblages, is macroscopically similar to Petrographic Fabric 1 but petrographically distinguished by a higher glauconite content. Nevertheless, it falls within the Palermitan chemical group (Geochemical Cluster 1), suggesting that it may also represent a Palermo production.

Outside Palermo, several macroscopic, petrographic, and geochemical groups can be identified at various sites, indicating ceramic production in workshops located outside Palermo. Seven macroscopic fabrics were initially identified and tentatively attributed to Agrigento (AGR1–2), Syracuse (SR1), Piazza Armerina (PZAR1), and, more tentatively, Mazara del Vallo (CDPT1–3), on the basis of previous literature which analysed wasters, kiln furniture, and on the assumption that clay sources were located near workshop areas. Petrographic analysis subsequently validated and revised this classification. In some cases, macroscopic and petrographic classifications corresponded closely (e.g. SR1 = Petrographic Fabric 2), whereas in others petrography demonstrated that apparently distinct macroscopic groups shared the same paste recipe, leading to their merging or reassignment (e.g. AGR1 and AGR2 = Petrographic Fabric 6; CDPT1–3 = Petrographic Fabric 7). These revisions indicate that the macroscopic classification overemphasised differences in colour and surface appearance, traits more likely related to firing conditions than to the use of different raw materials or paste recipes (Quinn 2022: 161). From a geochemical perspective, the ceramics suspected to be from the other sites, grouped mainly in Geochemical Clusters 2 and 4, display greater variability than the Palermitan wares of Geochemical Cluster 1, as also shown in the PCA (Fig. 5). While this pattern should be interpreted cautiously given the smaller sample sizes and the resulting reduced robustness of the statistical groupings, it may partly reflect a less homogeneous production background than that observed for the Palermitan material. Paste standardisation depends on multiple social and technological factors and does not necessarily correlate directly with workshop specialisation (Arnold 2000). Nevertheless, previous studies suggests that these production centres likely exploited locally available clay sources (Alaimo-Giarrusso 2007; Alfano et al. 2019; Ben Amara et al. 2009; Capelli et al. 2021).

The samples assigned to Petrographic Fabrics 2, 4, and 6, all interpreted as Sicilian products, fall within Geochemical Cluster 2. Only a few sherds assigned to Petrographic Fabrics 2 and 4 fall outside Geochemical Cluster 2, which may result from post-depositional alteration, or other sources of analytical variation. Although chemically grouped together, these fabrics remain petrographically distinct and form separate subclusters within the PCA and HCA analyses (Fig. 6). Petrographic Fabric 2, attributed to Syracuse, is characterised by abundant carbonate inclusions and volcanic lithics, features consistent with eastern Sicilian volcanic-derived ceramics previously described by Ben Amara et al. (2009). The presence of kiln rods among the associated finds further supports local manufacture. The regional geology, comprising marine, fluvial, and lacustrine sandy clays and gravels, calcareous tuffs, fossiliferous limestones, and loosely consolidated conglomerates and sands, corresponds well with the petrographic characteristics observed in this fabric.

In contrast, Petrographic Fabric 4, identified at Piazza Armerina, is calcareous and, also petrographically compatible with local geological formations (Alfano et al. 2019). The surrounding geology includes a wide range of clay deposits, marls, limestones, colluvial deposits, sandy fossiliferous sediments, bituminous clays, and mudstone breccias containing exotic lithic inclusions. The mineralogical variability observed in the fabric is compatible with this sedimentary complexity and supports the hypothesis of local production in the Villa del Casale area. Ceramic production at the site is archaeologically documented, further supporting a local attribution.

Further support for local production comes from Petrographic Fabric 6, represented by sherds sampled from *Fornaci S. Lucia* in Agrigento, whose characteristics corresponds well with both archaeological and archaeometric evidence for local production (Alaimo-Giarrusso 2007; Cuomo di Caprio 1992; 1995). The geology of the Agrigento area includes calcareous tuffs, shell breccias, fossiliferous marine clays, conglomerates with Eocene and Miocene pebbles, gypsum deposits, marine sands and gravels, and bituminous clays, all of which provide plausible sources for the inclusions and mineralogical characteristics observed in the fabric. The sherds were recovered near kiln remains and production waste, reinforcing this interpretation.

The remaining Sicilian petrographic fabrics, Fabrics 5 and 7, do not correspond to single geochemical groups. Petrographic Fabric 5 occurs in Geochemical Clusters 2 and 4, while Petrographic Fabric 7 is distributed across Geochemical Clusters 2, 3 and 4, making their provenance more difficult to interpret. This suggests the use of chemically comparable clay sources or significant overlap between Sicilian and North African paste recipes, although it should also be considered in light of the smaller sample sizes and the resulting reduced robustness of the statistical groupings. Petrographic Fabric 5 has tentatively been interpreted as being produced at Piazza Armerina, whereas Petrographic Fabric 7 has been attributed to Mazara del Vallo (Capelli et al. 2021). However, macroscopic, petrographic, and geochemical analyses consistently demonstrate the difficulty of distinguishing these ceramics from other paste recipes. Their shared petrographic characteristics, including calcareous to moderately calcareous matrices with dominant quartz inclusions, may reflect either the use of similar ceramic production practices and raw material preparation techniques, or the exploitation of comparable geological resources. From a petrographic perspective, Petrographic Fabric 7 is compatible with the geology of the Mazara area, particularly the Marsala and Belice formations (Di Geronimo et al. 1994; Regione Sicilia – VIGOR 2013; Capelli et al. 2021). These formations include recent alluvial deposits, coastal dunes, detrital-bioclastic limestones, tuffaceous-calcareous conglomerates, marls rich in Globigerina, gypsum-bearing deposits, fossiliferous clays, shell breccias, and sandy clays containing foraminifera and molluscs.

Ceramics tentatively attributed to Ifriqiyan production (Petrographic Fabrics 8–11) cluster mainly within Geochemical Cluster 3 and therefore show a degree of compositional consistency, although some samples assigned to Petrographic Fabric 11 fall outside this group. The petrographic, macroscopic, and chemical classifications do not show a straightforward one-to-one correspondence, which is not unexpected given the small sample size and the compositional similarity of the calcareous paste recipes represented in the corpus. More generally, the petrographic differentiation of Ifriqiyan productions is hindered by the homogeneity of inclusion types and the broadly calcareous or highly calcareous nature of the ceramic matrices. Variability is expressed primarily through differences in inclusion density, granulometry, and distribution. Although the samples tentatively attributed to Ifriqiyan production and corresponding to Geochemical Cluster 3 tend to cluster together, their distribution in the PCA remains dispersed rather than tightly cohesive. Overall, the lack of strong coherence, particularly within Petrographic Fabrics 9 and 11, highlights the difficulty of confidently assigning precise provenance or isolating the signature of specific clay sources without a substantially larger comparative dataset. Comparison with previously published analyses of Ifriqiyan glazed wares, especially those from the Medjerda Valley analysed by Occari et al. (2025) and the Ifriqiyan imports studied by Waksman (2024), did not resolve these issues definitively (Fig. 8). For this reason, future work will expand the analytical dataset in order to better characterise the different production centres involved.

Finally, sample VSC025, classified as Fabric 12, constitutes a clear outlier within the dataset. Previous petrographic analyses conducted by Capelli (2024), together with comparison to reference groups, suggest an Egyptian origin based on petrographic similarities with established Egyptian reference groups. This interpretation is further supported by the typology of the vessel, specifically a splashed ware characterised by a glaze applied over a slip layer.

## Conclusions

The results of this study confirm the existence of multiple glazed ware production centres operating across the island, each associated with distinct compositional traditions. Palermo was the earliest and most long-lived production centre (late 9th – 11th century) during the Islamic period, characterised by the use of the Ficarazzi clay and a highly consistent paste recipe. Beyond Palermo, the combined macroscopic, petrographic, geochemical, and archaeological evidence supports the existence of additional glazed ware productions probably located at Syracuse, Piazza Armerina, Agrigento, and Mazara del Vallo, which appear to have developed from the late 10th century onwards, although the evidence remains uneven in strength. In contrast to the more coherent compositional pattern identified for Palermitan wares, the other production groups display greater variability in paste composition. This can be related to their smaller sample sizes, although it cannot be excluded that the observed variability may point to a broader range of technological choices and raw material sources among these production centres. The integration of petrographic and geochemical data proved particularly effective in distinguishing Palermitan wares from North African imports, despite the strong stylistic similarities that have long complicated their attribution. Nevertheless, the results also highlight the difficulty of separating certain Sicilian and Ifriqiyan calcareous fabrics, especially those tentatively associated with Mazara and some North African products. This may reflect technological convergence, shared production traditions, or the exploitation of geologically comparable clay sources across the region.

Shared technological features, including high firing temperatures and the widespread use of salt-white surface treatments on iron-rich fabrics, point to the circulation of common technical knowledge between the different production centres in Sicily, and between Sicily and Ifriqiya. These similarities may reflect the mobility of artisans, the transmission of specialised skills, or broader networks of technological exchange.

By establishing preliminary petrographic and geochemical groups for Sicilian glazed wares, this study provides a framework for future provenance research and for the identification of Sicilian products at consumption sites within and beyond the island. Expanding the comparative dataset, particularly for North African products and currently underrepresented Sicilian workshops, will be essential for refining these reference groups and for further reconstructing patterns of production, exchange, and technological interaction in the medieval central Mediterranean.

## Supplementary Information

Below is the link to the electronic supplementary material.

Supplementary Material 1

Supplementary Material 2

Supplementary Material 3

Supplementary Material 4

Supplementary Material 5

## Data Availability

All data supporting the findings of this study are available within the paper and its Supplementary Information.
