# Supplementary material for: Tracing glazed ware production in Islamic Sicily: macroscopic, petrographic, and geochemical evidence
Source: Archaeol Anthropol Sci. 2026 Sep 2;18(9):195. doi: 10.1007/s12520-026-02550-1 (PMC13538216; doi:10.1007/s12520-026-02550-1)
Supplement: Supplementary file 1 [file 12520_2026_2550_MOESM1_ESM.docx]

| Macro-fabric | Fracture Description | Void Frequency & Type | Inclusions | Samples |
| --- | --- | --- | --- | --- |
| PA1 | Rough to the touch, red, medium-high hardness and compactness | Low frequency, irregularly shaped | White (high freq., small–medium, often perforated); Black (rare, small); Transparent (low freq., small, angular–rounded) | GA1-11; GA15-GA18; GA20; GA22-23; GA25-26; GA28-38; GA42-43; GA47-55; GA57; GA59-63; GA66-70; GA74-90; GA92; PB1-2; PB4-13; PB16-25; PB27; PB29-32; PZA8?, PZA11 |
| PA2 | Rough to the touch, dark grey, medium-high hardness and compactness | Low frequency, irregularly shaped | Same as PA1; differs only in body colour | GA12; GA14; GA19; GA21; GA24; GA27; GA39; GA41; GA44; GA56; GA58; GA64; GA72—73; GA91; PB3; PB15; PB26 |
| PA3 | Fracture fairly smooth to the touch, red/orange in colour, medium-high hardness and compactness | Low frequency, with irregular shapes | White (medium-high frequency, small to medium size); transparent (medium frequency, angular and rounded shapes). Overall appearance is similar to PA1 and PA2 but with fewer white inclusions, which are not perforated in the center | GA71; GA93 |
| IFR5 | Well-cleaned, smooth, white/beige, high hardness, medium-high compactness | Medium frequency (small round); medium-low frequency (medium-sized) | White (low freq., small–medium); Transparent (low freq., small–medium) | GA97; GA105. |
| IFR8 | Powdery to the touch, white/beige, medium hardness and compactness | Medium frequency, irregularly shaped | White (low freq., small); Transparent (high freq., small, angular–rounded) | GA102; GA104; GA107; GA108; GA109; PB34. |
| IFR16 | Rough to the touch, light pink, medium hardness, medium-high compactness | Low frequency, irregularly shaped | White (medium-low freq., small); Black (rare, small); Transparent (high freq., small–medium, angular–rounded) | GA94; GA95; GA98; GA99; GA103; GA106; GA110; GA111; PB33. |
| IFR46 | Fracture fairly smooth to the touch, light pink, medium- high hardness and compactness | Medium-law frequency, irregularly shaped | White (medium freq., small); Black (rare, small); Transparent (medium-high freq., small, angular-rounded) | GA96 |
| IFR64 | Smooth to the touch, light pink, high hardness and compactness | Low frequency, small, irregularly shaped | White (low freq., small, sometimes perforated); Transparent (medium-high freq., small, angular–rounded) | GA101. |
| IFR89 | Rough to the touch, light pink center (Munsell 2.5YR 8/4), light grey surfaces (Munsell 5Y 8/2) | Low frequency, small and medium-sized | Transparent (high freq., small–medium, rounded); Black (rare, small) | PV6? |
| EG47 | Rough to the touch, pink (Munsell 5YR 8/4), medium-high hardness and compactness | Medium-low frequency, small, irregularly shaped | White (medium-high freq., small); Transparent (medium freq., small, angular) | GA100. |
| SR1 | Rough to the touch, red near surface, brown/dark red core, medium-high hardness and compactness | Low frequency, irregular and elongated | White (high freq., medium, sometimes perforated); Transparent (rare, medium); Fine gold (few) | SIR1; SIR2; SIR3; SIR4; SIR5; SIR6; SIR7; SIR8; SIR9; SIR10; SIR11; SIR12. |
| AGR1 | Rough to the touch, red near surface, pale red or red core with greenish patches, medium hardness and compactness | Low frequency, irregular and elongated | White (medium freq., small–medium, often perforated); Transparent (low–medium freq., small–medium); Black (rare, medium); Fine gold (medium freq.) | AG2; AG3; AG4; AG5; AG6; AG8; AG9; AG10. |
| AGR2 | Rough to the touch, red near surface, pale red with greenish patches, medium hardness and compactness | Low frequency, irregular and elongated | White (medium freq., small–medium, often perforated); Transparent (low–medium freq., small–medium); Fine gold (medium freq.) **Note**: Same fabric as AGR1 with a different firing | AG1; AG7. |
| PZAR1 | Rough and slightly powdery to the touch, beige to pink, low hardness, medium compactness | Medium frequency, irregular and elongated | Transparent (high freq., small–medium); White (rare, medium); Black (rare, small); Red (rare–low, small); Fine gold (low–medium freq.) | PZA1; PZA2?; PZA3; PZA4; PZA5; PZA6; PZA7; PZA9; PZA10; PZA12; PZA13; PZA14; PZA15; PZA16. |
| CDPT1 | Rough to the touch, beige in the core and close to the surfaces, pink with red stripes in the rest | Low to medium frequency, small, irregularly shaped, very few elongated | Transparent (high freq., small–medium, rounded); white (low freq., medium size some with a void inside); red (rare, medium and big size); grey (low freq., medium size) | CDP3 |
| CDPT2 | Rough to the touch, light pink/grey in the core, red and then beige close to the surface | Medium freq., elongated and irregular | Transparent (high freq., small–medium, rounded); white (medium freq., medium size some with a void inside); black (low freq., medium size) | CDP2 |
| CDPT3 | Rough to the touch, beige, pink or dark pink with a thin beige layer close to one of the surfaces | Medium freq. of irregular, low freq. of elongated | Transparent (high freq., small–medium, rounded); white (medium freq., medium size some with a void inside); red (rare, medium and big size); | CDP1; CDP4?; CDP5; CDP6. |
