# Supplementary material for: Tracing glazed ware production in Islamic Sicily: macroscopic, petrographic, and geochemical evidence
Source: Archaeol Anthropol Sci. 2026 Sep 2;18(9):195. doi: 10.1007/s12520-026-02550-1 (PMC13538216; doi:10.1007/s12520-026-02550-1)
Supplement: Supplementary file 2 [file 12520_2026_2550_MOESM2_ESM.docx]

Supplementary Material 4: Petrographic descriptions

1. **Ficarazzi clay**

Samples: GA1, GA2, GA3, GA4, GA5, GA6, GA7, GA8, GA9, GA11, GA12, GA14, GA15, GA16, GA17, GA18, GA19, GA20, GA21, GA22, GA23, GA24, GA25, GA26, GA27, GA28, GA29, GA30, GA31, GA32, GA34, GA35, GA36, GA37, GA38, GA39, GA41, GA42, GA43, GA44, GA45, GA47, GA48, GA49, GA50, GA52, GA53, GA54, GA55, GA56, GA57, GA58, GA60, GA61, GA63, GA64, GA66, GA67, GA68, GA69, GA70, GA71, GA72, GA73, GA74, GA75, GA77, GA78, GA79, GA80 (very few patches), GA81, GA82, GA83, GA84, GA85, GA86, GA87, GA88, GA89, GA90, GA91, GA92, GA93, PB1, PB2, PB3, PB4, PB6, PB7, PB9, PB11, PB12, PB15, PB16, PB17, PB18, PB19, PB20, PB21, PB23, PB24, PB25, PB26, PB27, PB29, PB30, PB31, PB32, PV1, PV2, PV3, PV5, PZA11.

**Inclusions**

20-30%. Eq. & el., sr. & sa., closed spaced to single spaced, poorly sorted, randomly oriented.

*Dominant*: mainly Monocrystalline and few Polycrystalline Quartz; mainly eq. and few el., sr. & sa., single spaced, poorly aligned, 0.025 to 0.2mm (mode 2mm).

*Frequent to Dominant*: Micritic Limestone; 0.05 to 0.25mm (mode 0.1mm), sometimes surrounded by olive-colour halo. Samples display different grade of degradation of calcite.

*Common*: Ferruginous nodules; eq., sa. & sr., 0.05 to 0.3mm (mode 0.2mm).

*Few*: Chert; el. & eq., sa., 0.06 to 0.5mm, sometimes infilled with iron (cf. PB31).

*Rare*: Bioclasts (cf. benthic foraminifera in samples GA72 and GA81, microfossil in sample GA16); 0.4 to 0.75mm (mode 0.5mm). Sample GA15 has a higher number of shells.

*Very rare to very few*: Glauconite; eq. (rare to absent el.), sr., 0.1 to 0.35mm (mode 0.2mm). Samples GA12, GA19, GA20 (?), GA43, GA48, GA60, GA63, GA91, PB9, PV2, PV5 contain more glauconite.

*Very rare*: Biotite; flakes (0.05mm) possible eq., sr. in sample GA53.

*Very rare*: Muscovite; flakes (0.15mm) and possible eq., sa. in samples GA17, GA72, GA53.

*Very rare to absent*: Clinopyroxene (GA7); sa., eq., 0.15mm.

*Very rare to absent*: Plagioclase feldspar (GA70, GA77, PB3); eq., sa., 0.1 to 0.15mm.

*Very rare to absent*: Conglomerate of Quarts (GA88) and a sort of lump of iron rich clay with quartz and one muscovite or olivine (GA88).

*Very rare to absent*: Ferruginous nodules with quartz inside (GA88, GA30, PB31, PB21, GA54, GA37, GA44, GA79, GA66).

**Matrix**

60%. All samples are optically inactive and display varying color tones depending on the firing conditions. The most common type is a heterogeneous matrix that appears red in both PPL and XP. This matrix is characterized by more or less evident greenish halos, which result from reactions related to the decomposition of limestone and salts (GA1, GA2, GA3, GA5, GA6, GA7, GA8, GA9, GA11, GA15, GA16, GA17, GA18, GA20, GA22, GA23, GA24, GA25, GA26, GA27, GA28, GA32, GA34, GA35, GA36, GA38, GA39, GA42, GA43, GA45, GA47, GA48, GA49, GA50, GA52, GA53, GA54, GA55, GA57, GA60, GA61, GA63, GA67, GA68, GA69, GA70, GA74, GA75, GA77, GA79, GA80, GA81, GA83, GA84, GA85, GA87, GA88, GA89, GA90, PB1, PB4, PB6, PB7, PB9, PB11, PB12, PB17, PB18, PB19, PB20, PB21, PB23, PB24, PB25, PB27, PB29, PB30, PB31, PB32, PV1, PV2, PV3, PV5, PZA11). A darker brown variant of this heterogeneous fabric is also observed, typically showing olive-colored patches of varying extent. This includes samples GA12, GA14, GA19, GA21, GA29, GA30, GA31, GA41, GA44, GA58, GA64, GA72, GA73, GA91, PB3, PB15, and PB26. The other two variants of heterogeneous matrix are represented by sample GA66, which has a light orange core and red close to the edges, with olive halos, and by sample PB16, which is light orange with olive patches. A small number of samples exhibit a moderately red, homogeneous matrix under both PPL and XP conditions. These include GA4, GA71, GA78, GA82, GA86, GA92, GA93 and PB2. Only six samples have a truly homogeneous matrix, with colors ranging from olive (GA56), to brown (GA30, GA37), and dark brown (GA31, GA91, PB26). Nearly all samples exhibit one or two lighter-colored surfaces, typically olive-toned, with a thickness ranging from 0.25 mm to 1.5 mm. Some samples, such as PB18, GA70, GA71, GA72, GA86, GA93, and PV1, appear to lack these lighter surfaces—although in some cases this may be due to the matrix itself already being largely olive in color. Finally, some samples are characterized by patches of secondary calcite (GA3, GA8, GA15, GA16, GA21, GA29, GA32, GA36, GA38, GA57, GA60, GA61, GA64, GA68, GA75, GA87, GA89, PB6, PB17, PB20, PB27, PB31, PB32).

**Voids**

10-20%. Meso vesicles and and meso- and macro-vughs, mainly surrounded by a greenish edge. Sometimes they are partially infilled with secondary calcite in the internal edge. Some samples are characterized by some few elongate voids, sometimes infilled with secondary calcite (GA14, GA15, GA54, GA20, GA23, GA60, GA61, PB31), sometimes interpretable as the trace of plant matter (GA24, GA35, GA45, GA63, GA55, GA56, PB1, PB30).

**Comments**

The Ficarazzi clay group is characterized by a non-calcareous, optically inactive fabric with a high inclusion density (20–30%), comprising predominantly monocrystalline quartz and frequent micritic limestone, often displaying varying degrees of calcite degradation. Additional inclusions include ferruginous nodules, chert, and rare bioclasts (notably mollusks and foraminifera), along with trace amounts of glauconite, biotite, muscovite, feldspar, and iron-rich clay aggregates.

The matrix (~60%) is typically heterogeneous and red in both PPL and XP, often displaying greenish or olive halos associated with the decomposition of limestone and salts. These halos, which frequently occur around voids or calcite inclusions, are chromatically similar to the vitrified lighter surfaces observed on most samples (0.25–1.5 mm thick). This correlation suggests the addition of salt to the paste, which likely migrated during drying and locally fluxed the clay minerals, initiating a reaction with the calcite inclusions. In contrast, homogeneous matrices—though less common—tend to show no or minimal calcite degradation, indicating greater thermal or chemical stability under firing conditions.

Voids are present in moderate abundance (10–20%) and consist mainly of meso-vesicles and meso- to macro-vughs, frequently with greenish rims and occasionally infilled with secondary calcite. Elongate voids, some possibly related to decomposed plant matter, are also observed. Importantly, a lower abundance of voids generally coincides with preserved calcite, supporting the interpretation that many voids result from calcite decomposition during firing. Patches of secondary calcite are common, reflecting post-depositional or firing-induced reprecipitation.

2. **Calcite and Altered Mafic Tempered Fabric**

Samples: SIR1, SIR2, SIR3, SIR4, SIR6, SIR7, SIR8, SIR9.

**Inclusions**

30%. Eq. & el., sr.; closed spaced to open-spaced. Randomly oriented. Bimodal grain size distribution, poorly sorted.

Coarse fraction

40-50%. 0.1mm to 0.75mm

*Dominant*: Limestone; mostly micritic, occasionally sparry lacking allochems; eq. & el., sr., 0.1mm to 0.75mm (mode 0.35mm), moderately to poorly sorted, closed spaced to open spaced, poorly aligned. The coarse sand-sized calcite is not uniformly distributed throughout the section. Some limestone inclusions show iron staining. In some samples (SIR2, SIR6, SIR7) the micritic limestone inclusions have broken down during firing to leave voids.

*Few to very rare*: Mica; mode 0.15mm. Almost absent in SIR9 and SIR1. In sample SIR8, the biotite mica is mainly present close to the edges.

*Very few*: Mafic weathered material; eq. & el., r., 0.1-0.35mm (mode 2mm). Orange to red with fibrous structure. Composed of alteration product such as iddingsite or oxidized serpentine. Usually surrounded by a void.

*Very few*: Bioclasts; eq. & el., sr. & sa., 0.25 to 0.55mm (mode 0.4mm).

*Rare*: Plagioclase feldspar; el., sa., 0.1mm to 0.45mm (mode 0.25mm).

*Rare*: Quartz, mainly monocrystalline and rare polycrystalline (SIR2); eq. & el., sa, 0.15-0.3mm (mode 0.2mm). In sample SIR6 there is a bit more quartz, sometimes iron stained.

*Rare*: Ferruginous nodules; eq., r., 0.1 to 0.25mm (mode 0.15mm).

*Very rare to absent*: Chert; eq., sr. 0.45mm (SIR1 and SIR11).

*Very rare to absent*: possible Glauconite (SIR7); eq., sr., 0.1mm.

*Very rare to absent*: Altered basic igneous rock fragments cf. basalt; eq., sr., 0.025 to 0.5mm. Composed of elongate mineral laths of possible feldspar aligned in a particular direction in a brown weathered groundmass (SIR2 and SIR9).

*Very rare to absent*: Amphibole. Orange red coloured due to oxidation; el., sr., 0.2mm (SIR3 and SIR2).

*Very rare to absent*: Clinopyroxene; eq., sr., 0.25mm (SIR9 and SIR2).

Fine fraction

50-60%. <0.1mm.

- *Dominant*: micritic Calcite.
- *Few*: Quartz.
- *Few*: Ferruginous nodules.

**Matrix**

60%. Non-calcareous matrix, which can vary in colour due to firing condition. Samples SIR1, SIR4 and SIR9 are brown in the core and orange at the edges in PPL; brown/grey in the core and red/orange at the edges in XP. The matrix is relatively homogeneous but may contain lighter orange stripes parallel to the edges and darker brown areas in the core. The orange part shows very low activity, while the core is optically inactive. SIR3 is characterized by a brown core, transitioning to red and then back to brown near the edges. Samples SIR2, SIR6 and SIR8 are optically inactive with a homogeneous matrix, brown/red in PPL, reddish brown in XP (SIR2), dark grey in both PPL and XP in SIR6 and SIR8. All samples have some olive coloured patches (0.3 to 0.9mm) of the same colour of the layer visible beneath the glaze (ranging from 0.05 to 0.3mm) in all samples and that often surround also some limestone inclusions and voids.

**Voids**

10-20%. Consisting of meso-vesicles, and macro- and rare mega-vughs. Many of the vughs are associated with degraded calcite, sometimes surrounded by a calcareous greenish fringe (SIR2, SIR6 and SIR7). Samples SIR2, SIR6, SIR7 and SIR8 have more abundant meso-vesicles and vughs, compatible with the degradation of the calcite component in limestone and bioclasts. Elongate voids infilled with secondary calcite, strongly aligned with the edges are visible in samples SIR1, SIR4 and SIR9.

**Comments**

The dominance of micritic limestone and the presence of weathered mafic material are the two main characteristics of this fabric. The samples exhibit variation in firing conditions, as suggested by differences in the matrix colour and the state of calcite preservation. Samples SIR1, SIR3, SIR4, and SIR9 were fired to a lower temperature and therefore the original calcareous inclusions are more intact. In contrast, samples SIR2, SIR6, SIR7, SIR8 have more abundant meso-vesicles and vughs surrounded by a greenish edge, which is a feature consistent with calcite degradation, and appear to reflect higher firing temperatures. Secondary calcite deposition after burial is also observed in some samples (SIR1, SIR4, and SIR9), typically within elongate voids.

The non-uniform distribution of coarse, sand-sized calcite suggests that artisans may have added compositionally diverse sand temper—containing eroded limestone, shells, and mafic igneous material—to a non-calcareous clay source.

Finally, the greenish layer beneath the glaze of all samples can be interpreted as a salt-white surface. This fabric is clearly distinguished from the other fabrics in this study by the dominant presence of micritic limestone and the presence of weathered mafic material, and less quartz inclusions.

3. **Calcite and Glauconite Fabric**

Samples: SIR10, SIR11, SIR12.

**Inclusions**

30%. Mainly eq., few el., closed-spaced to single-spaced. Randomly oriented, moderately sorted.

*Dominant*: Micritic Limestone; eq. & el., sr. & sa. 0.05 to 0.35mm (mode 0.2mm). In sample SIR10 the grain size of limestone is smaller (mode 0.05mm), moderately sorted, poorly aligned. In samples SIR11 and SIR12, the larger micritic limestone inclusions have broken down during firing to leave voids.

*Frequent*: Monocrystalline Quartz; eq., sa. & sr., 0.05 to 0.4mm (mode 0.15mm).

*Common*: Glauconite; eq., sr. & sa. 0.05 to 0.2mm (mode 0.1mm).

*Few to common*: Biotite Mica; eq. & sr., 0.05 to 0.15mm (mode 0.1mm).

*Few*: Ferruginous nodules; eq., sr., 0.05 to 0.25mm (mode 0.1mm).

*Very few*: Chert; eq., sa., 0.2 to 0.3mm (mode 0.25mm).

*Very rare*: possible Muscovite Mica.

*Very rare*: Bioclasts.

**Matrix**

60%. Non-calcareous matrix, red with olive-coloured patches from the decomposition of calcite and its reaction with the clay matrix and probably the salt contained in PPL and XP. Heterogeneous, optically inactive.

**Voids**

10%. Meso-vesicles and meso- and macro-vughs. The vughs are associated with degraded calcite, sometimes surrounded by a greenish calcareous fringe. Sample SIR10 has no macro-vughs.

**Comments**

The main characteristics of this fabric are the use of calcite temper and the frequent presence of glauconite. The three samples are similar, although sample SIR10 lacks macro-vughs and generally contains smaller calcite inclusions.

While the frequency of quartz inclusions is slightly higher than in Fabric 2, it remains relatively low compared to the rest of the corpus. This feature, along with the use of calcite temper, may suggest a relationship with Fabric 2. However, the presence of glauconite and the absence of mafic material distinguish these samples from Fabric 2.

4. **Biotite-rich Calcareous Sandy Quartz Fabric**

Samples: PZA1, PZA2, PZA3, PZA4, PZA5, PZA7, PZA10, PZA12, PZA13, PZA16.

**Inclusions**

20%-30%. Eq. & el., sa., close-spaced to single spaced. Randomly oriented, moderately sorted.

*Predominant-Dominant*: Monocrystalline Quartz; eq. & e., sa. 0.025 to 0.45mm (mode 0.15mm). Very few Polycrystalline Quartz. Unimodal presence of quartz (sometimes weakly bimodal) (very-fine sand size and medium sand size). Occasionally iron-stained (e.g. PZA1).

*Common*: Ferruginous nodules; eq. & el., sr. & sa.; 0.025 – 1mm (mode 0.2mm).

*Few*: Biotite; el. & eq., sr., 0.06 to 0.14mm (mode 0.1mm). In sample PZA1 the flakes of biotite are particularly abundant close to the edges.

*Rare*: Chert; eq., sr., 0.06 to 0.1mm (mode 0.08mm).

*Rare*: Muscovite; el. & eq., 0.15 – 0.2mm (mode 0.1mm). Sample PZA5 is characterized by more flakes of muscovite, close to the edges.

*Very rare*: Plagioclase Feldspar; eq., sa., 0.1 to 0.25mm (mode 0.2mm).

*Very rare*: Microcline Feldspar; eq., sr. & sa., 0.25 to 0.8mm (mode 0.15mm).

*Very rare*: Bioclasts, eq., sr., 0.25mm.

**Matrix**

60-70%. Calcareous. Orange/yellow in PPL, orange/greenish in XP. Homogeneous. Optically inactive. In samples PZA1, PZA2, PZA5, PZA13 localized iron-rich staining have been identified. Samples PZA7, PZA10, PZA12, PZA13 and PZA16 are characterized by patches of secondary calcite.

**Voids**

10%. Meso - vesicles and vughs, rare macro-vughs, very rare mega vughs. Some exhibit secondary calcite at the margins.

**Comments**

This homogeneous highly calcareous fabric is characterized by the abundant sand-sized quartz and biotite mica, which are sometimes concentrated along the edges of the samples. The quartz inclusions exhibit a unimodal grain size distribution, ranging from very fine to medium sand. However, the distribution of quartz in the coarse fraction suggests the addition of quartzose sand temper. The matrix is uniform and occasionally marked by patches of secondary calcite (e.g., PZA7, PZA10, PZA12, PZA13, PZA16).

Although the nature of inclusions is common – particularly among calcareous fabrics – the grain size of quartz, combined with the high abundance of biotite mica and the occasional presence of microcline feldspar, distinguishes this fabric from the others in this study.

5. **Calcareous Silty Quartz Fabric**

Samples: PZA6, PZA9, PZA14, PZA15.

**Inclusions**

20%. Eq. &el., sa. &sa., randomly oriented, moderately well sorted.

*Predominant*: Monocrystalline Quartz; mainly eq., few el., mainly sa., few sr., 0.025 to 0.75mm (mode 0.05mm). Rare polycrystalline quartz. In sample PZA9 the sand-sized quartz fraction is higher.

*Rare*: Ferruginous inclusions; eq., sr., 0.05mm.

*Very rare to very few*: biotite mica, el., rare eq., sr., 0.05mm.

*Very rare*: Chert; eq. & el., sa., 0.2mm.

*Very rare*: Microcline Feldspar; eq., sa., 0.15 to 0.45mm (mode 0.3mm).

*Very rare*: Plagioclase Feldspar; eq., sa., 0.05 to 0.25mm (PZA9, PZA15).

**Matrix**

70%. Calcareous, buff in PPL, olive in XP. Optically inactive, strongly homogeneous, with some patches of secondary calcite.

**Voids**

10%. Micro-vesicles, meso-vesicles and vughs, rare macro-vughs. Few elongate voids aligned with the vessel margins.

**Comments**

The silty grain size of the quartz is the main characteristic of this calcareous fabric. The presence of a lump of matrix without inclusions in sample PZA6 may suggest that the quartz was added as temper. In many aspects, this fabric is similar to Fabric 4; however, the lower presence of biotite mica distinguishes the two. Although a relationship between them cannot be excluded, the differences in mica content suggest some variation in raw material or preparation techniques.

6. **Biotite Rich Non-calcareous Silty Quartz Fabric**

Samples: AG1, AG2, AG3, AG4, AG5, AG6, AG7, AG8, AG9, AG10.

**Inclusions**

20-30%. El. & eq., sa., single-spaced to double-spaced. Randomly oriented, poorly sorted.

*Dominant*: Monocrystalline Quartz; eq. & el., sa. & sr., 0.05 to 0.25mm (mode 0.15mm), poorly sorted. In samples AG1 and AG3 silty sized grains are more abundant.

*Frequent*: Micritic limestone; el. & eq., sr., 0.1 to 0.45mm (mode 0.25mm).

*Few to common*: Biotite mica; el. & eq., sr., 0.025 to 0.08mm (mode 0.06mm).

*Few*: Ferruginous inclusions; eq., sr., 0.025 to 0.35mm (mode 0.1mm).

*Rare*: Muscovite Mica; el. & sa., 0.08mm.

*Very few*: Bioclasts; eq. & el., sa. & sr., 0.1 to 0.3mm (mode 0.2mm). Some can be identified as foraminifera.

*Very rare*: Chert; eq., sa., 0.06 to 0.15mm (mode 0.1mm).

*Very rare*: Plagioclase Feldspar; eq., sr., 0.1mm.

*Very rare*: Feldspar; eq., sa., 0.08 to 0.1mm. Cf. Microcline.

*Very rare to absent*: Green birefringent mineral; eq., sa., 0.1mm. Cf. Amphibole (?). Sample AG10.
*Very rare to absent*: Sandy limestone containing quartz, biotite and possibly amphibole (AG2)

**Matrix**

60-70%. Heterogeneous and inactive matrix, orange in PPL and XP, with greenish patches in samples AG1, AG3, AG7, AG8, AG9, AG10. Slightly homogeneous and poorly active, orange/brown in the core, red close to the edges in PPL and XP in sample AG2; orange in PPL, red in XP in samples AG4, AG5 and AG6. In sample AG1 and AG3: the core is lighter compared to the edges, which are more reddish and in the second right beneath the reddish part is a stripe of secondary calcite.

In all cases just beneath the glaze, there is a greenish layer of 0.15 to 0.35 mm of thickness, which is thinner in AG4 and AG5. Localized iron-rich staining has been identified in sample AG10.

**Voids**

10%. Meso-vesicles and few meso- and macro-vughs. The vughs are compatible with the degradation of the calcite. In AG3 and AG7 the macro-vughs are more frequent, elongate and parallel orientation to the surfaces. Sample AG2 has elongate voids parallel to the vessel margins, which have formed due to the destruction of plant matter. AG5 has less voids (5%).

**Comments**

This homogeneous fabric is characterized by dominant quartz and frequent calcite, primarily in the form of limestone, along with a few bioclasts and microfossils. The inclusions are generally poorly sorted and randomly oriented, mostly ranging from coarse silt to fine sand in size. They include occasional to common biotite mica and ferruginous nodules. Subordinate inclusions identified are chert, plagioclase, and microcline feldspar.

The samples in this fabric differ in terms of the nature of their clay matrix, most likely resulting from differences in firing conditions. Some samples are characterized by greenish patches (e.g., AG5308, AG1, AG3, AG7, AG8, AG5376), while the others lack these features (e.g., AG2, AG4, AG5, AG6). Finally, the greenish layer beneath the glaze can be interpreted has a salt-white surface.

7. **Calcareous to moderately calcareous Fabric with Bimodal Quartz Inclusions (fine to medium sand size of the coarse fraction).**

Samples: CDP1, CDP2, CDP3, CDP4, CDP5, CDP6.

**Inclusions**

20%. Eq., sr. & sa.; closed spaced to open-spaced. Randomly oriented. Bimodal grain size distribution, very poorly sorted.

Coarse fraction

40 to 60%. 0.05mm to 0.5mm.

*Dominant*: Monocrystalline Quartz; eq., sr., 0.05mm to 0.5mm (mode 0.25mm), very few polycrystalline.

*Few*: Biotite Mica flakes (fewer in CDP3 and CDP4).

*Few*: Ferruginous nodules; eq, r. & sr., 0.05 to 0.2mm (0.1mm).

*Very rare*: Chert; 0.25mm eq. sr.

*Very rare*: Microcline Feldspar; eq., sr. 0.25mm (all except CDP1).

*Rare to absent:* Amphibole; eq., sr., 0.4mm (CDP4; CDP5).

*Rare to absent*: Plagioclase Feldspar; eq., sa., 0.3mm (CDP1).

Fine fraction

40 to 60%. < 0.05mm.

- Quartz.
- Biotite mica.

**Matrix**

60%. Inactive, heterogeneous, some olive patches in XP dark orange; PPL buff and brown (CDP1, CDP5, CDP6); olive in the core and close to the edges, two red stripes with secondary calcite same in PPL (CDP2). Poorly active) Olive with red stripes here and there (CDP3).

Patches of secondary calcite in all samples (except CDP5), sometimes aligned with the edges in stripes.

**Voids**

20%. Meso-vesicles, elongated strongly oriented with the edges, sometimes infilled with secondary calcite.

**Comments**

This fabric is characterized by a calcareous to moderately calcareous matrix with a distinct bimodal distribution of quartz inclusions, ranging from fine to medium sand sizes within the coarse fraction. The poorly sorted inclusions are predominantly monocrystalline quartz, accompanied by minor amounts of biotite mica flakes, ferruginous nodules, and trace accessory minerals such as chert, microcline, amphibole, and plagioclase. The matrix is generally inactive and heterogeneous, showing variable coloration including buff, brown, and olive patches, often with secondary calcite aligned in stripes, particularly evident in samples CDP2 and CDP3. Voids account for approximately 20% of the fabric, presenting as meso-vesicles that are elongated and strongly oriented near the edges, frequently infilled with secondary calcite, indicative of post-depositional alteration. Compared to the similar bimodal fabric group 9.1, this fabric contains increased amounts of biotite and microcline, reflecting subtle compositional differences. The variation in bimodal grain size distribution suggests diverse sedimentary inputs or processing techniques during manufacture.

8. **Highly Calcareous Fabric with Sparce Quartz**

Samples: GA97; GA105.

**Inclusions**

3%. Eq., sa., open spaced. Randomly oriented, poorly sorted.

*Dominant*: Monocrystalline Quartz; eq. and few el., sr. & sa., 0.025 to 0.35mm (mode 0.1mm), poorly sorted.

*Frequent*: Ferruginous inclusions; 0.025 to 0.5mm (mode 0.025mm).

*Rare to absent*: Microcline Feldspar; 0.2mm (GA852).

**Matrix**

95%. Calcareous, homogeneous and inactive, buff in PPL, greenish/yellow in XP.

**Voids**

2%. Meso vesicles and vughs, micro vesicles.

**Comments**

This fabric, represented by two samples, is characterized by a low inclusion content and a highly calcareous matrix, showing minimal signs of post-depositional alteration. While the inclusions—primarily quartz and ferruginous nodules—are similar to those found in other fabrics (Fabrics 7, 9, 10 and 11), this one is clearly distinguished by their limited quantity and sparse distribution.

9. **Moderately Calcareous Sandy Quartz Fabric**

**Moderately Calcareous Sandy Quartz Subfabric 1**

Samples: CDP7, GA108, PV6.

**Inclusions**

20-30%. Eq., sr. & sa.; closed spaced to single-spaced. Randomly oriented. Bimodal grain size distribution, poorly sorted.

Coarse fraction

60%. 0.1mm to 0.75mm (mode 0.3mm).

*Predominant*: Monocrystalline Quartz mainly; eq., sr. & sa., 0.05 to 0.65mm (mode 0.3mm); rare Polycrystalline quartz.

*Few*: Ferruginous nodules; eq. & el., sr., 0.15 to 0.75mm (mode 0.25mm).

*Rare*: Chert; eq., sr., 0.15 to 0.35mm.

*Rare to absent*: Plagioclase Feldspar; eq., sr. & sa., 0.15mm (GA108, PV6).

Fine fraction

30%. <0.05mm.

- Quartz
- Ferruginous nodules

**Matrix**

60-70%. Moderately calcareous matrix, inactive, buff in PPL, greenish/yellow in XP, homogeneous in sample CDP7, heterogeneous in GA108 and PV6 which are characterized by pale red patches. We can observe patches of secondary calcite in sample PV6.

**Voids**

10%. Meso-vesicles mainly and few macro-vughs; elongate voids strongly aligned with the margins.

**Moderately Calcareous Sandy Quartz Subfabric 2**

Samples: GA96, GA102, GA104, GA106, GA110, PB28, PV8.

**Inclusions**

20-30%. Eq., sr. & sa.; closed spaced to single-spaced. Randomly oriented. Bimodal grain size distribution, poorly sorted.

Coarse fraction

70%. 0.1 to 0.3mm (mode 0.2mm)

*Predominant*: Monocrystalline Quartz mainly; eq., sr. & sa., 0.05 to 0.5mm (mode 2mm); rare Polycrystalline.

*Very Few*: Ferruginous nodules; eq. & el., sr., 0.05 to 0.15mm (mode 0.05mm). More has been observed in sample PB28.

*Rare*: Chert; eq., sr., 0.15 to 0.5mm.

*Rare to absent*: Biotite Mica (GA96, GA110).

*Rare to absent*: Plagioclase Feldspar; eq., sr. & sa., 0.1mm (GA110).

Fine fraction

30%. <0.05 mm.

- Quartz.
- Ferruginous nodules. In sample PV8 the ferruginous nodules are more abundant.

**Matrix**

60-70%. Moderately calcareous fabric, optically inactive (very low optical activity only in sample GA102). It exhibits variability in homogeneity. Samples PV8, GA106, and GA110 display a heterogeneous fabric, characterized by an orange core and olive-colored edges observable in both PPL and XP. GA110 also shows olive patches within the orange areas under XP, further highlighting localized alteration. In contrast, the remaining samples present a homogeneous appearance, showing a buff tone in PPL and olive in XP. Secondary calcite patches are noted in GA104, GA106, and GA96, suggesting post-depositional calcite development.

**Voids**

10%. Meso-vesicles, very few macro-vughs (GA110, PB28, PV8) some with secondary calcite in the internal edges (GA110).

**Comments**

The bimodal nature of this fabric suggests that a moderately calcareous clay was tempered with sand. However, variations in the degree of bimodality among the inclusions led to the division into two subgroups. Subgroup 1 is distinctly bimodal, with a coarse fraction ranging from fine to medium sand. An exception is sample GA108, which appears less bimodal and contains a finer-grained coarse fraction. In contrast, Subgroup 2 shows weaker bimodality, with a coarse fraction ranging from very fine to fine sand. Another distinguishing feature is the presence of elongate voids in Subgroup 1, which are absent or extremely rare in Subgroup 2.

10. **Calcite and Quartz-Tempered Calcareous Fabric**

Sample: PV7.

**Inclusions**

30%. Mainly eq., few el., closed-spaced to single-spaced. Randomly oriented. Bimodal grain size distribution.

Coarse fraction

70%. 0.15 to 0.55mm (mode 0.25mm).

*Dominant*: Monocrystalline Quartz; eq., sa. & sr., 0.15 to 0.55mm (mode 0.25mm), moderately sorted, closed spaced to single spaced.

*Frequent*: Micritic limestone and bioclasts (cf. Orbullina sp.); eq. & el., r. & sr. Concentrated in specific part of the thin section.

*Rare*: Chert; eq., sa., 0.2 to 0.3mm (mode 0.25mm).

Rare to absent: Birefringent inclusion (cf. Amphibole); eq., sr. 0.2mm.

Fine fraction

30%. <0.05 mm.

- Quartz.
- Ferruginous nodules.

**Matrix**

50-60%. Moderately calcareous, olive/orange in PPL and XP, heterogeneous, some areas are more orange than others. Optically poorly active.

**Voids**

10-20%. Meso-vesicles and vughs, few macro-vughs, elongate voids infilled with secondary calcite.

**Comments:**

This unique sample closely resembles Fabric 9, particularly Subgroup 1, due to its strong bimodality and similar inclusion characteristics. However, it differs in containing frequent to dominant micritic limestone and bioclasts, indicating the addition of material from a distinct sand deposit as temper. Additionally, it is marked by the presence of secondary calcite within elongate voids. While it is possible that the same clay source was used as in Fabric 9, the differences suggest a different technological choice during production.

11. **Slightly Calcareous Fine Sandy Quartz Fabric**

Samples: GA94, GA95, GA98, GA99, GA101, GA103, GA107, GA109, GA111, PB33, PB34, PV4.

**Inclusions**

40%. Eq., sr. & sa., single-spaced, randomly oriented, moderately sorted.

*Predominant*: Monocrystalline Quartz; eq., sr. & sa., 0.025 to 0.75mm (mode 0.2mm). Very few el. Very few with iron stain and polycrystalline quartz.

*Common*: Ferruginous nodules; 0.025 to 0.25mm (mode 0.1mm).

*Very Rare*: Biotite Mica (absent in samples GA107 and GA101); possible eq., 0.1mm to 0.2mm (GA94, GA861, PV4). Sample PV4 contains more Biotite.

*Rare to absent*: Chert; eq., sa., 0.16mm (PB33, GA99, GA101, GA111); sometimes with iron stain.

*Rare to absent*: Feldspar (cf. Microcline); eq, sa., 0.04 to 0.3mm (GA94, GA95, GA103 with iron stain).

*Rare to absent*: Feldspar (cf. Plagioclase); eq., sa., 0.06mm.

*Rare to absent*: Highly Birefringent inclusion (cf. Muscovite Mica); el., 0.12mm.

Rare to absent: Zircon; eq., 0.04mm (GA95).

Rare to absent: Micritic Limestone; eq., sr., 0.35mm (GA99, PB34). More in PB34.

**Matrix**

Most samples are heterogeneous, appearing orange in plane-polarized light (PPL) and pale red in cross-polarized light (XP), with samples GA95, GA98, and GA103 showing distinctive olive patches. Two exceptions are noted: GA107, which is homogeneous and appears dark yellow to brownish in XP, and GA11, which shows a pale brown hue in XP. All samples—except GA107—exhibit an olive-colored layer beneath the glaze, with variable thickness. Additionally, samples GA35, GA109, and PB391 display patches of secondary calcite.

**Voids**

10-20%. Meso vesicles and vughs. More voids in PB33. Very few elongate voids (PB33 and GA103), elongate voids with infilled secondary calcite well orientated to the edges (GA111, PB34).

**Comments**

This fabric group is characterized by a weakly calcareous, fine sandy quartz matrix with a relatively high inclusion content (~40%), dominated by well-sorted, equant, subrounded to subangular monocrystalline quartz grains. The matrix is generally heterogeneous, displaying warm tones in both PPL and XP, with several samples (e.g., GA95, GA98, GA103) exhibiting distinct olive patches, possibly reflecting thermal variability. Notably, the consistent presence of an olive layer beneath the glaze—except in GA107—may indicate the use of a salt-white surface technique. Voids are present primarily as meso-vesicles and vughs, with some infilled and oriented along the margins, pointing to both original porosity and secondary processes such as calcite precipitation (e.g., GA111, PB391).
