# Supplementary material for: Tracing glazed ware production in Islamic Sicily: macroscopic, petrographic, and geochemical evidence
Source: Archaeol Anthropol Sci. 2026 Sep 2;18(9):195. doi: 10.1007/s12520-026-02550-1 (PMC13538216; doi:10.1007/s12520-026-02550-1)
Supplement: Supplementary file 3 [file 12520_2026_2550_MOESM4_ESM.docx]

**Supplementary materials 1**

| **Site** | **SU** | **Sample n.** | **INAA n.** | **Type/Subtype** | **Pottery chronology** | **Macroscopic Fabric** | **Petrographic Fabric** | **Geochemistry Group** | **Provenance** |
| --- | --- | --- | --- | --- | --- | --- | --- | --- | --- |
| Gancia | I462 | GA1 | VSC119 | YELL | 10^th^-11^th^ century | PA1 | 1 | Cluster 1 | Palermo |
| Gancia | F169 | GA2 | VSC067 | YELL | First half of the 11^th^ | PA1 | 1 | Cluster 1 | Palermo |
| Gancia | H296 | GA3 | VSC071 | YELL | Second half of the 10^th^ | PA1 | 1 | Cluster 1 | Palermo |
| Gancia | C52 | GA4 | VSC093 | YELL | 10^th^-11^th^ century | PA1 | 1 | Cluster 1 | Palermo |
| Gancia | H307 | GA5 | VSC097 | YELL | First half of the 10^th^ | PA1 | 1 | Cluster 1 | Palermo |
| Gancia | L349 | GA6 | - | YELLPNT | 10^th^-11^th^ century | PA1 | 1 | N.A. | Palermo |
| Gancia | G0 | GA7 | VSC136 | YELLPNT | 10^th^-11^th^ century | PA1 | 1 | Cluster 1 | Palermo |
| Gancia | G264 | GA8 | VSC064 | YELLPNT/Palermo yellow | Late 9^th^ - early 10^th^ | PA1 | 1 | Cluster 1 | Palermo |
| Gancia | G Trincea | GA9 | VSC134 | YELLPNT/Palermo yellow | 10^th^-11^th^ century | PA1 | 1 | Cluster 1 | Palermo |
| Gancia | G264 | GA11 | - | YELLPNT/Palermo yellow | Late 9^th^ - early 10^th^ | PA1 | 1 | N.A. | Palermo |
| Gancia | G264 | GA12 | VSC104 | YELLPNT/Palermo yellow | Late 9^th^ - early 10^th^ | PA2 | 1 | Cluster 1 | Palermo |
| Gancia | C0 | GA14 | VSC143 | TRANSPARENT UNCOLOURED | 10^th^-11^th^ century | PA2 | 1 | Cluster 1 | Palermo |
| Gancia | L427 | GA15 | VSC144 | TRANSPARENT UNCOLOURED | 10^th^-11^th^ century | PA1 | 1 | Cluster 1 | Palermo |
| Gancia | L461 | GA16 | VSC140 | TRANSPARENT UNCOLOURED | 10^th^-11^th^ century | PA1 | 1 | Cluster 1 | Palermo |
| Gancia | L427 | GA17 | VSC145 | POLYCH/OPA? | 10^th^-11^th^ century | PA1 | 1 | Cluster 1 | Palermo |
| Gancia | H315 | GA18 | VSC102 | POLYCH | First half of the 10^th^ | PA1 | 1 | Cluster 1 | Palermo |
| Gancia | B54 | GA19 | - | POLYCH | 10^th^-11^th^ century | PA2 | 1 | N.A. | Palermo |
| Gancia | B54 | GA20 | - | POLYCH | 10^th^-11^th^ century | PA1 | 1 | N.A. | Palermo |
| Gancia | L438 | GA21 | VSC098 | POLYCH | Second half of the 10^th^ | PA2 | 1 | Cluster 1 | Palermo |
| Gancia | L430 | GA22 | VSC149 | POLYCH | 10^th^ century | PA1 | 1 | Cluster 1 | Palermo |
| Gancia | L430 | GA23 | - | POLYCH | 10^th^ century | PA1 | 1 | N.A. | Palermo |
| Gancia | L348 | GA24 | - | POLYCH | 10^th^-11^th^ century | PA2 | 1 | N.A. | Palermo |
| Gancia | L418 | GA25 | VSC122 | POLYCH | 10^th^-11^th^ century | PA1 | 1 | Cluster 1 | Palermo |
| Gancia | C380 | GA26 | VSC099 | POLYCH | Late 10^th^ - early 11^th^ | PA1 | 1 | Cluster 1 | Palermo |
| Gancia | C380 | GA27 | VSC109 | POLYCH | Late 10^th^ - early 11^th^ | PA2 | 1 | Cluster 1 | Palermo |
| Gancia | C398 | GA28 | VSC120 | POLYCH | 10^th^-11^th^ century | PA1 | 1 | Cluster 1 | Palermo |
| Gancia | F169 | GA29 | VSC085 | POLYCH | First half of the 11^th^ | PA1 | 1 | Cluster 1 | Palermo |
| Gancia | F179 | GA30 | VSC138 | POLYCH | 10^th^-11^th^ century | PA1 | 1 | Cluster 1 | Palermo |
| Gancia | L456 | GA31 | VSC084 | POLYCH | Late 9^th^ - early 10^th^ | PA1 | 1 | Cluster 1 | Palermo |
| Gancia | L438 | GA32 | VSC086 | POLYCH | Second half of the 10^th^ | PA1 | 1 | Cluster 1 | Palermo |
| Gancia | L430 | GA34 | VSC133 | POLYCH | 10^th^ century | PA1 | 1 | Cluster 1 | Palermo |
| Gancia | L418 | GA35 | VSC137 | POLYCH | 10^th^-11^th^ century | PA1 | 1 | Cluster 1 | Palermo |
| Gancia | L418 | GA36 | - | POLYCH | 10^th^-11^th^ century | PA1 | 1 | N.A. | Palermo |
| Gancia | C380 | GA37 | VSC062 | POLYCH | Late 10^th^ - early 11^th^ | PA1 | 1 | Cluster 1 | Palermo |
| Gancia | H307 | GA38 | VSC070 | POLYCH | First half of the 10^th^ | PA1 | 1 | Cluster 4 | Palermo |
| Gancia | H307 | GA39 | VSC101 | POLYCH | First half of the 10^th^ | PA2 | 1 | Cluster 1 | Palermo |
| Gancia | C238 | GA41 | VSC132 | POLYCH/graticcio | 10^th^-11^th^ century | PA2 | 1 | Cluster 1 | Palermo |
| Gancia | C380 | GA42 | VSC103 | POLYCH/graticcio | Late 10^th^ - early 11^th^ | PA1 | 1 | Cluster 1 | Palermo |
| Gancia | L438 | GA43 | VSC095 | POLYCH/graticcio | Second half of the 10^th^ | PA1 | 1 | Cluster 1 | Palermo |
| Gancia | B54 | GA44 | - | POLYCH/graticcio | 10^th^-11^th^ century | PA2 | 1 | N.A. | Palermo |
| Gancia | H300 | GA45 | VSC072 | POLYCH/graticcio | Second half of the 10^th^ | PA1 | 1 | Cluster 1 | Palermo |
| Gancia | C249 | GA47 | VSC090 | POLYCH/splash | Late 10^th^ - early 11^th^ | PA1 | 1 | Cluster 1 | Palermo |
| Gancia | F169 | GA48 | VSC105 | POLYCH/splash | First half of the 11^th^ | PA1 | 1 | Cluster 1 | Palermo |
| Gancia | F168 | GA49 | VSC092 | POLYCH/splash | Late 10^th^ – first half of the 11^th^ | PA1 | 1 | Cluster 1 | Palermo |
| Gancia | L430 | GA50 | VSC088 | POLYCH | 10^th^ century | PA1 | 1 | Cluster 1 | Palermo |
| Gancia | B54 | GA51 | VSC130 | POLYCH | 10^th^-11^th^ century | PA1 | N.A. | Cluster 1 | Palermo |
| Gancia | C380 | GA52 | VSC089 | POLYCH | Late 10^th^ - early 11^th^ | PA1 | 1 | Cluster 1 | Palermo |
| Gancia | A22 | GA53 | VSC115 | POLYCH | 10^th^-11^th^ century | PA1 | 1 | Cluster 1 | Palermo |
| Gancia | A19 | GA54 | VSC139 | POLYCH | 10^th^-11^th^ century | PA1 | 1 | Cluster 1 | Palermo |
| Gancia | L418 | GA55 | VSC118 | GREEN | 10^th^-11^th^ century | PA1 | 1 | Cluster 1 | Palermo |
| Gancia | C249 | GA56 | VSC094 | GREEN | Late 10^th^ - early 11^th^ | PA2 | 1 | Cluster 1 | Palermo |
| Gancia | L427 | GA57 | VSC124 | GREEN | 10^th^-11^th^ century | PA1 | 1 | Cluster 1 | Palermo |
| Gancia | H296 | GA58 | VSC110 | GREEN | Second half of the 10^th^ | PA2 | 1 | Cluster 1 | Palermo |
| Gancia | H296 | GA59 | VSC106 | GREEN | Second half of the 10^th^ | PA1 | N.A. | Cluster 1 | Palermo |
| Gancia | H309 | GA60 | - | GREEN | 10^th^-11^th^ century | PA1 | 1 | N.A. | Palermo |
| Gancia | B54 | GA61 | VSC148 | GREEN | 10^th^-11^th^ century | PA1 | 1 | Cluster 1 | Palermo |
| Gancia | C260 | GA62 | VSC117 | GREEN | 10^th^-11^th^ century | PA1 | N.A. | Cluster 1 | Palermo |
| Gancia | C380 | GA63 | VSC063 | GREEN | Late 10^th^ - early 11^th^ | PA1 | 1 | Cluster 1 | Palermo |
| Gancia | H283 | GA64 | VSC091 | GREEN | Late 10^th^ - early 11^th^ | PA2 | 1 | Cluster 1 | Palermo |
| Gancia | L339 | GA66 | - | GREENPNT | 10^th^-11^th^ century | PA1 | 1 | N.A. | Palermo |
| Gancia | L339 | GA67 | - | GREENPNT | 10^th^-11^th^ century | PA1 | 1 | N.A. | Palermo |
| Gancia | L418 | GA68 | VSC135 | GREENPNT | 10^th^-11^th^ century | PA1 | 1 | Cluster 1 | Palermo |
| Gancia | C380 | GA69 | VSC087 | GREENPNT | Late 10^th^ - early 11^th^ | PA1 | 1 | Cluster 1 | Palermo |
| Gancia | B54 | GA70 | VSC123 | GREENINC | 10^th^-11^th^ century | PA1 | 1 | Cluster 1 | Palermo |
| Gancia | L430 | GA71 | VSC073 | GREENINC | 10^th^ century | PA3 | 1 | Cluster 1 | Palermo |
| Gancia | H296 | GA72 | VSC082 | BROW | Second half of the 10^th^ | PA2 | 1 | Cluster 1 | Palermo |
| Gancia | C292 | GA73 | VSC141 | BROW | 10^th^-11^th^ century | PA2 | 1 | Cluster 1 | Palermo |
| Gancia | F167 | GA74 | VSC100 | BROW | 10^th^-12^th^ century | PA1 | 1 | Cluster 1 | Palermo |
| Gancia | F154 | GA75 | VSC068 | BROW | 10^th^-12^th^ century | PA1 | 1 | Cluster 1 | Palermo |
| Gancia | L438 | GA76 | - | OPA | Second half of the 10^th^ | PA1 | 1 | N.A. | Palermo |
| Gancia | B54 | GA77 | - | OPA | 10^th^-11^th^ century | PA1 | 1 | N.A. | Palermo |
| Gancia | L430 | GA78 | VSC065 | OPA | 10^th^ century | PA1 | 1 | Cluster 1 | Palermo |
| Gancia | L430 | GA79 | VSC074 | OPA | 10^th^ century | PA1 | 1 | Cluster 1 | Palermo |
| Gancia | L429 | GA80 | VSC121 | OPA | 10^th^-11^th^ century | PA1 | 1 | Cluster 1 | Palermo |
| Gancia | L339 | GA81 | VSC150 | OPA | 10^th^-11^th^ century | PA1 | 1 | Cluster 1 | Palermo |
| Gancia | F169 | GA82 | VSC083 | OPA/TDA | First half of the 11^th^ | PA1 | 1 | Cluster 1 | Palermo |
| Gancia | F169 | GA83 | VSC081 | OPA/TDA | First half of the 11^th^ | PA1 | 1 | Cluster 1 | Palermo |
| Gancia | F116 | GA84 | VSC125 | OPA/TDA | 10^th^-11^th^ century | PA1 | 1 | Cluster 1 | Palermo |
| Gancia | F113 | GA85 | VSC142 | OPA/TDA | 10^th^-11^th^ century | PA1 | 1 | Cluster 1 | Palermo |
| Gancia | F169 | GA86 | - | OPA/TDA | Late 10^th^ - early 11^th^ | PA1 | 1 | N.A. | Palermo |
| Gancia | L348 | GA87 | VSC128 | OPA/TDA | 10^th^-11^th^ century | PA1 | 1 | Cluster 1 | Palermo |
| Gancia | A22 | GA88 | VSC066 | TUR | 10^th^-11^th^ century | PA1 | 1 | Cluster 1 | Palermo |
| Gancia | H283 | GA89 | VSC127 | TUR | Late 10^th^ - early 11^th^ | PA1 | 1 | Cluster 1 | Palermo |
| Gancia | B29 | GA90 | VSC126 | TUR | 10^th^-11^th^ century | PA1 | 1 | Cluster 1 | Palermo |
| Gancia | F169 | GA91 | VSC069 | OVERGLAZED | First half of the 11^th^ | PA2 | 1 | Cluster 1 | Palermo |
| Gancia | L339 | GA92 | VSC131 | MOULD | 10^th^-11^th^ century | PA1 | 1 | N.A. | Palermo |
| Gancia | L438 | GA93 | VSC108 | GREENINC | Second half of the 10^th^ | PA3 | N.A. | Cluster 1 | Palermo |
| Gancia | G264 | GA94 | - | YELLPNT/Raqqada yellow | Late 9^th^ - early 10^th^ | IFR16 | 11 | N.A. | Ifriqiya |
| Gancia | L438 | GA95 | - | YELLPNT | Second half of the 10^th^ | IFR16 | 11 | N.A. | Ifriqiya |
| Gancia | C380 | GA96 | VSC146 | OPA? | Late 10^th^ - early 11^th^ | IFR46 | 9.2 | Cluster 3 | Ifriqiya |
| Gancia | I415 | GA97 | VSC022 | YELL | 10^th^-11^th^ century | IFR5 | 8 | Cluster 3 | Ifriqiya |
| Gancia | G264 | GA98 | VSC021 | YELLPNT | Late 9^th^ - early 10^th^ | IFR16 | 11 | Cluster 3 | Ifriqiya |
| Gancia | I462 | GA99 | VSC020 | YELLPNT/Raqqada yellow | 10^th^-11^th^ century | IFR16 | 11 | Cluster 3 | Ifriqiya |
| Gancia | C380 | GA100 | VSC025 | POLYCH/splash | Late 10^th^ - early 11^th^ | EG47 | 12 | Outlier | Egypt |
| Gancia | C264 | GA101 | VSC024 | YELLPNT | Late 9^th^ - early 10^th^ | IFR64 | 11 | Outlier | Ifriqiya |
| Gancia | H283 | GA102 | - | YELLPNT | Late 10^th^ - early 11^th^ | IFR8 | 9.2 | N.A. | Ifriqiya |
| Gancia | L438 | GA103 | VSC019 | POLYCH | Second half of the 10^th^ | IFR16 | 11 | Cluster 3 | Ifriqiya |
| Gancia | G327 | GA104 | - | POLYCH | Late 9^th^ - early 10^th^ | IFR8 | 9.2 | N.A. | Ifriqiya |
| Gancia | C380 | GA105 | VSC002 | TUR? | Late 10^th^ - early 11^th^ | IFR5 | 8 | Cluster 3 | Ifriqiya |
| Gancia | B54 | GA106 | VSC003 | TUR? | 10^th^-11^th^ century | IFR16 | 9.2 | Cluster 3 | Ifriqiya |
| Gancia | L456 | GA107 | - | YELLPNT | Late 10^th^ - early 11^th^ | IFR8 | 11 | N.A. | Ifriqiya |
| Gancia | C380 | GA108 | VSC034 | POLYCH/Splash? | Late 10^th^ - early 11^th^ | IFR8 | 9.1 | Cluster 3 | Ifriqiya |
| Gancia | C164 | GA109 | VSC018 | POLYCH | 10^th^-11^th^ century | IFR8 | 11 | Cluster 3 | Ifriqiya |
| Gancia | C380 | GA110 | VSC001 | INCISED | Late 10^th^ - early 11^th^ | IFR16 | 9.2 | Cluster 3 | Ifriqiya |
| Gancia | F155 | GA111 | VSC023 | YELLPNT | 10^th^ - 11^th^ century | IFR16 | 11 | Outlier | Ifriqiya |
|  |  |  |  |  |  |  |  |  |  |
| Palazzo Bonagia | 31 2B | PB1 | - | YELL | Late 10^th^ - early 11^th^ | PA1 | 1 | N.A. | Palermo |
| Palazzo Bonagia | 71 2A | PB2 | - | YELL | 10^th^ century | PA1 | 1 | N.A. | Palermo |
| Palazzo Bonagia | 31 2A | PB3 | - | YELLPNT | 10^th^ century | PA2 | 1 | N.A. | Palermo |
| Palazzo Bonagia | 97 3A | PB4 | VSC129 | YELLPNT/Palermo yellow | Late 9^th^ - early 10^th^ | PA1 | 1 | Cluster 1 | Palermo |
| Palazzo Bonagia | 65 2B | PB6 | VSC057 | POLYCH | Late 10^th^ - early 11^th^ | PA1 | 1 | Cluster 1 | Palermo |
| Palazzo Bonagia | 64 2C | PB7 | VSC075 | POLYCH | Second half of the 10^th^ | PA1 | 1 | Cluster 1 | Palermo |
| Palazzo Bonagia | 34 1C | PB9 | VSC112 | POLYCH | Late 10^th^ - early 11^th^ | PA1 | 1 | Cluster 1 | Palermo |
| Palazzo Bonagia | 31 3C | PB11 | VSC059 | POLYCH/graticcio | Late 10^th^ - early 11^th^ | PA1 | 1 | Cluster 1 | Palermo |
| Palazzo Bonagia | 41 2A | PB12 | VSC061 | POLYCH/graticcio | Late 10^th^ - early 11^th^ | PA1 | 1 | Cluster 1 | Palermo |
| Palazzo Bonagia | 65 2B | PB13 | - | POLYCH/graticcio | Late 10^th^ - early 11^th^ | PA1 | 1 | N.A. | Palermo |
| Palazzo Bonagia | 36 2C | PB15 | VSC077 | POLYCH/graticcio | Late 10^th^ - early 11^th^ | PA2 | 1 | Cluster 1 | Palermo |
| Palazzo Bonagia | - | PB16 | - | POLYCH/graticcio | 10^th^ century | PA1 | 1 | N.A. | Palermo |
| Palazzo Bonagia | 65 2B | PB17 | VSC078 | POLYCH/graticcio | Late 10^th^ - early 11^th^ | PA1 | 1 | Cluster 1 | Palermo |
| Palazzo Bonagia | 120 3C | PB18 | VSC111 | POLYCH/splash | Late 10^th^ - early 11^th^ | PA1 | 1 | Cluster 1 | Palermo |
| Palazzo Bonagia | 36 2C | PB19 | - | POLYCH/splash | Late 10^th^ - early 11^th^ | PA1 | 1 | N.A. | Palermo |
| Palazzo Bonagia | 36 2C | PB20 | VSC114 | POLYCH | Late 10^th^ - early 11^th^ | PA1 | 1 | Cluster 1 | Palermo |
| Palazzo Bonagia | 36 2C | PB21 | VSC060 | POLYCH | Late 10^th^ - early 11^th^ | PA1 | 1 | Cluster 1 | Palermo |
| Palazzo Bonagia | 65 2B | PB22 | VSC116 | POLYCH | Late 10^th^ - early 11^th^ | PA1 | N.A. | Cluster 4 | Palermo |
| Palazzo Bonagia | 36 2C | PB23 | VSC056 | POLYCH | Late 10^th^ - early 11^th^ | PA1 | 1 | Cluster 1 | Palermo |
| Palazzo Bonagia | 65 2B | PB24 | VSC076 | GREEN | Late 10^th^ - early 11^th^ | PA1 | 1 | Cluster 1 | Palermo |
| Palazzo Bonagia | 31 1A | PB25 | VSC080 | GREEN | 10^th^-11^th^ century | PA1 | 1 | Cluster 1 | Palermo |
| Palazzo Bonagia | 31 1A | PB26 | - | GREEN | 10^th^-11^th^ century | PA2 | 1 | N.A. | Palermo |
| Palazzo Bonagia | 31 3C | PB27 | VSC107 | OPA | Late 10^th^ - early 11^th^ | PA1 | 1 | Cluster 1 | Palermo |
| Palazzo Bonagia | 31 2B | PB28 | VSC005 | OPA | Late 10^th^ - early 11^th^ | IFR | 9.2 | Cluster 4 | Ifriqiya |
| Palazzo Bonagia | 38 2C | PB29 | VSC058 | OPA | 10^th^ century | PA1 | 1 | Cluster 1 | Palermo |
| Palazzo Bonagia | 31 1A | PB30 | - | OPA | 10^th^-11^th^ century | PA1 | 1 | N.A. | Palermo |
| Palazzo Bonagia | 93 3B | PB31 | VSC113 | OPA | 10^th^-11^th^ century | PA1 | N.A. | Cluster 4 | Palermo |
| Palazzo Bonagia | 34 1C | PB32 | VSC079 | OPA/TDA | Late 10^th^ - early 11^th^ | PA1 | 1 | Cluster 1 | Palermo |
| Palazzo Bonagia | 31 1A | PB33 | VSC055 | YELLPNT | 10^th^-11^th^ century | IFR16 | 11 | Cluster 3 | Ifriqiya |
| Palazzo Bonagia | 65 2B | PB34 | VSC004 | POLYCH? | Late 10^th^ - early 11^th^ | IFR8 | 11 | Outlier | Ifriqiya |
|  |  |  |  |  |  |  |  |  |  |
| Piazza della Vittoria | - | PV1 | - | YELLPNT | 10^th^-11^th^ century | PA1 | N.A. | N.A. | Palermo |
| Piazza della Vittoria | - | PV2 | - | YELLST | 10^th^-11^th^ century | PA1 | N.A. | N.A. | Palermo |
| Piazza della Vittoria | - | PV3 | VSC147 | BROW | 10^th^-11^th^ century | PA1 | N.A. | N.A. | Palermo |
| Piazza della Vittoria | - | PV4 | VSC096 | TUR | 10^th^-11^th^ century | N.A. | 11 | Cluster 3 | Ifriqiya |
| Piazza della Vittoria | - | PV5 | - | CUERDA | 10^th^-11^th^ century | PA1 | 1 | N.A. | Palermo |
| Piazza della Vittoria | - | PV6 | VSC006 | OPAQUE | 10^th^-11^th^ century | IFR89? | 9.1 | Cluster 4 | Ifriqiya |
| Piazza della Vittoria | - | PV7 | - | TUR | 10^th^-11^th^ century | IFR | 10 | N.A. | Ifriqiya |
| Piazza della Vittoria | - | PV8 | VSC007 | TUR | 10^th^-11^th^ century | IFR | 9.2 | Cluster 3 | Ifriqiya |
|  |  |  |  |  |  |  |  |  |  |
| Castello della Pietra | - | CDP1 | VSC026 | TUR/Boli | 11^th^ century | CDPT3 | 7 | Cluster 3 | Mazara or Ifriqiya |
| Castello della Pietra | - | CDP2 | VSC011 | OPA/TDA | 11^th^ century | CDPT2 | 7 | Cluster 2 | Mazara |
| Castello della Pietra | - | CDP3 | VSC012 | TUR/Boli | 11^th^ century | CDPT1 | 7 | Cluster 4 | Mazara |
| Castello della Pietra | - | CDP4 | VSC010 | GREEN | 11^th^ century | CDPT3? | 7 | Cluster 4 | Mazara |
| Castello della Pietra | - | CDP5 | VSC008 | OPA/TDA | 11^th^ century | CDPT3 | 7 | Cluster 2 | Mazara |
| Castello della Pietra | - | CDP6 | VSC009 | OPA/TDA | 11^th^ century | CDPT3 | 7 | Cluster 2 | Mazara |
| Castello della Pietra | - | CDP7 | VSC013 | POLYCH | 11^th^ century | CDPT3 or IFR16 | 9.1 | Cluster 4 | Mazara or Ifriqiya |
|  |  |  |  |  |  |  |  |  |  |
| Agrigento | - | AG1 | VSC015 | GREENBROWN | First half of the 12^th^ | AGR2 | 6 | Cluster 2 | Agrigento |
| Agrigento | - | AG2 | VSC040 | POLYCH | First half of the 12^th^ | AGR1 | 6 | Cluster 2 | Agrigento |
| Agrigento | - | AG3 | VSC051 | GREENBROWN | First half of the 12^th^ | AGR1 | 6 | Cluster 2 | Agrigento |
| Agrigento | - | AG4 | VSC052 | POLYCH | First half of the 12^th^ | AGR1 | 6 | Cluster 2 | Agrigento |
| Agrigento | - | AG5 | VSC041 | POLYCH | First half of the 12^th^ | AGR1 | 6 | Cluster 2 | Agrigento |
| Agrigento | - | AG6 | VSC042 | POLYCH | First half of the 12^th^ | AGR1 | 6 | Cluster 2 | Agrigento |
| Agrigento | - | AG7 | VSC014 | POLYCH | First half of the 12^th^ | AGR2 | 6 | Cluster 2 | Agrigento |
| Agrigento | - | AG8 | VSC043 | POLYCH | First half of the 12^th^ | AGR1 | 6 | Cluster 2 | Agrigento |
| Agrigento | - | AG9 | - | POLYCH | First half of the 12^th^ | AGR1 | 6 | N.A. | Agrigento |
| Agrigento | - | AG10 | VSC016 | POLYCH | First half of the 12^th^ | AGR1 | 6 | Cluster 2 | Agrigento |
|  |  |  |  |  |  |  |  |  |  |
| Piazza Armerina | - | PZA1 | VSC032 | POLYCH | Late 10^th^ – first half of the 11^th^ | PZAR1 | 4 | Cluster 2 | Piazza Armerina |
| Piazza Armerina | - | PZA2 | VSC044 | OPA/TDA | Late 10^th^ – first half of the 11^th^ | PZAR1 or IFR? | 4 | Cluster 2 | Ifriqiya? |
| Piazza Armerina | - | PZA3 | VSC045 | OPA/TDA | Late 10^th^ – first half of the 11^th^ | PZAR1 | 4 | Cluster 2 | Piazza Armerina |
| Piazza Armerina | - | PZA4 | VSC046 | OPA/TDA | Late 10^th^ – first half of the 11^th^ | PZAR1 | 4 | Cluster 2 | Piazza Armerina |
| Piazza Armerina | - | PZA5 | VSC050 | POLYCH/SPLASH | Late 10^th^ – first half of the 11^th^ | PZAR1 | 4 | Cluster 2 | Piazza Armerina |
| Piazza Armerina | 1782 | PZA6 | VSC030 | POLYCH/SPLASH | Late 10^th^ – first half of the 11^th^ | PZAR1 | 5 | Cluster 4 | Piazza Armerina |
| Piazza Armerina | 1782 | PZA7 | VSC047 | OPA/TDA | Late 10^th^ – first half of the 11^th^ | PZAR1 | 4 | Outlier | Piazza Armerina? |
| Piazza Armerina | 5048 | PZA8 | VSC029 | OPA/TDA | Late 10^th^ – first half of the 11^th^ | PA1? | 1? | Cluster 2 | Palermo? |
| Piazza Armerina | 1782 | PZA9 | VSC033 | GREEN | Late 10^th^ – first half of the 11^th^ | PZAR1 | 5 | Cluster 2 | Piazza Armerina |
| Piazza Armerina | 1782 | PZA10 | - | OPA/TDA | Late 10^th^ – first half of the 11^th^ | PZAR1 | 4 | N.A. | Piazza Armerina |
| Piazza Armerina | 1782 | PZA11 | VSC028 | OPA/TDA | Late 10^th^ – first half of the 11^th^ | PA1? | 1 | Cluster 2 | Palermo? |
| Piazza Armerina | 1782=1967 | PZA12 | VSC049 | GREEN | Late 10^th^ – first half of the 11^th^ | PZAR1 | 4 | Cluster 4 | Piazza Armerina |
| Piazza Armerina | 1782=1967 | PZA13 | VSC017 | OPA/TDA | Late 10^th^ – first half of the 11^th^ | PZAR1 | 4 | Cluster 2 | Piazza Armerina |
| Piazza Armerina | 1782 | PZA14 | VSC031 | GREEN | Late 10^th^ – first half of the 11^th^ | PZAR1 | 5 | Cluster 4 | Piazza Armerina |
| Piazza Armerina | 1782 | PZA15 | VSC027 | TUR | Late 10^th^ – first half of the 11^th^ | PZAR1 | 5 | Cluster 4 | Piazza Armerina |
| Piazza Armerina | 1782=1967 | PZA16 | VSC048 | BROW | Late 10^th^ – first half of the 11^th^ | PZAR1 | 4 | Outlier | Piazza Armerina |
|  |  |  |  |  |  |  |  |  |  |
| Syracuse | - | SIR1 | VSC038 | OPA/TDA | Late 10^th^ – first half of the 11^th^ | SR1 | 2 | Cluster 2 | Syracuse |
| Syracuse | - | SIR2 | VSC035 | OPA/TDA | Late 10^th^ – first half of the 11^th^ | SR1 | 2 | Cluster 3 | Syracuse |
| Syracuse | - | SIR3 | VSC037 | OPA/TDA | Late 10^th^ – first half of the 11^th^ | SR1 | 2 | Cluster 2 | Syracuse |
| Syracuse | - | SIR4 | - | OPA/TDA | Late 10^th^ – first half of the 11^th^ | SR1 | 2 | N.A. | Syracuse |
| Syracuse | - | SIR5 | VSC039 | OPA/TDA | Late 10^th^ – first half of the 11^th^ | SR1 | 2 | Cluster 2 | Syracuse |
| Syracuse | - | SIR6 | - | OPA/TDA | Late 10^th^ – first half of the 11^th^ | SR1 | 2 | N.A. | Syracuse |
| Syracuse | - | SIR7 | VSC036 | OPA/TDA | Late 10^th^ – first half of the 11^th^ | SR1 | 2 | Cluster 2 | Syracuse |
| Syracuse | - | SIR8 | VSC054 | OPA/TDA | Late 10^th^ – first half of the 11^th^ | SR1 | 2 | Cluster 2 | Syracuse |
| Syracuse | - | SIR9 | - | OPA/TDA | Late 10^th^ – first half of the 11^th^ | SR1 | 2 | N.A. | Syracuse |
| Syracuse | - | SIR10 | VSC053 | OPA/TDA | Late 10^th^ – first half of the 11^th^ | SR1 | 3 | Cluster 1 | Palermo? |
| Syracuse | - | SIR11 | - | OPA/TDA | Late 10^th^ – first half of the 11^th^ | SR1 | 3 | N.A. | Syracuse |
| Syracuse | - | SIR12 | - | OPA/TDA | Late 10^th^ – first half of the 11^th^ | SR1 | 3 | N.A. | Syracuse |

**Legend**

BROW = Monochrome brown transparent ware

GREEN = Monochrome green transparent glaze

GREENBROWN = brown decoration under a green glaze

GREENINC = Monochrome green transparent glaze on one side, uncoloured transparent glaze on the other

GREENPNT = Polychrome decoration under a green transparent glaze

INCISED = monochrome glaze with an incised decoration

MOULD = monochrome glaze on a moulded ware

OPA = semi-opaque

OPA/TDA = semi-opaque, D’Angelo type ware

OPAQUE = opaque glazed ware

OVERGLAZED = polychrome decoration over an opaque white ware

POLYCH = Painted decoration under a transparent uncoloured glaze

POLYCH/graticcio = Painted decoration under a transparent uncoloured glaze, graticcio ware type

POLYCH/splash = Painted decoration under a transparent uncoloured glaze, splashed ware type

TRANSPARENT UNCOLOURED = Transparent uncoloured glaze

TUR = Monocrome green/turquoise opaque ware

TUR/Boli = Polychrome decoration over a turquoise opaque ware, *boli gialli* type

YELL= Transparent Yellow Glaze

YELLPNT = Painted decoration under a transparent yellow glaze

YELLPNT/Palermo yellow = Painted decoration under a transparent yellow glaze, Palermo yellow type

YELLPNT/Raqqada yellow = Painted decoration under a transparent yellow glaze, Raqqada yellow type
